# Michael Addition of 3-Oxo-3-phenylpropanenitrile to Linear Conjugated Enynones: Approach to Polyfunctional δ-Diketones as Precursors for Heterocycle Synthesis

**DOI:** 10.3390/molecules27041256

**Published:** 2022-02-13

**Authors:** Anastasiya V. Igushkina, Alexander A. Golovanov, Aleksander V. Vasilyev

**Affiliations:** 1 Department of Organic Chemistry, Institute of Chemistry, Saint Petersburg State University, Universitetskaya nab., 7/9, 199034 Saint Petersburg, Russia; kuznetsova.happy@yandex.ru; 2 S.P. Korshunov Research Laboratory No. 13, Department Chemical Technology and Resource Conservation, Togliatti State University, Belorusskaya ul., 14, 445667 Togliatti, Russia; aleksandgolovanov@yandex.ru; 3 Department of Chemistry, Saint Petersburg State Forest Technical University, Institutsky per., 5, 194021 Saint Petersburg, Russia

**Keywords:** conjugated enynones, 3-oxo-3-phenylpropanenitrile, Michael addition, δ-diketones, 1,2-diazepine

## Abstract

Reaction of linear conjugated enynones, 1,5-diarylpent-2-en-4-yn-1-ones [Ar^1^C≡CCH=CHC(=O)Ar^2^], with 3-oxo-3-phenylpropanenitrile (NCCH_2_COPh) in the presence of sodium methoxide MeONa as a base in MeOH at room temperature for 4–26 h affords polyfunctional δ-diketones as a product of regioselective Michael addition to the double carbon–carbon bond of starting enynones. The δ-diketones have been formed as mixtures of two diastereomers in a ratio of 2.5:1 in good general yields of 53–98%. A synthetic potential of the obtained δ-diketones has been demonstrated by heterocyclization with hydrazine into substututed 5,6-dihydro-4*H*-1,2-diazepine.

## 1. Introduction

Linear conjugated enynones, pent-2-en-4-yn-1-ones, are widely used as versatile building blocks in organic synthesis. The presence of three important functional groups, such as a carbonyl one, and double carbon–carbon and triple bonds, in their structure makes these compounds valuable precursors for synthesis of various polyfunctional compounds, and carbocycles, and, especially, heterocycles (see recent reviews on this issue [1,2]).

In our previous works [3,4], we studied multicomponent reactions of linear conjugated enynones, 1,5-diarylpent-2-en-4-yn-1-ones, with malononitrile as a CH-acid in the presence of different bases. Depending on the base used, these reactions gave rise to various products. In the case of using sodium alkoxide, as a strong nucleophilic base, substituted pyridines were obtained [3]. Contrary to that, a use of less nucleophilic base, lithium diisopropylamide (LDA), resulted in the formation of substituted cyclohexane as a major reaction product and Michael addition product to the double carbon–carbon bond as a minor one [4].

**Scheme 1:**
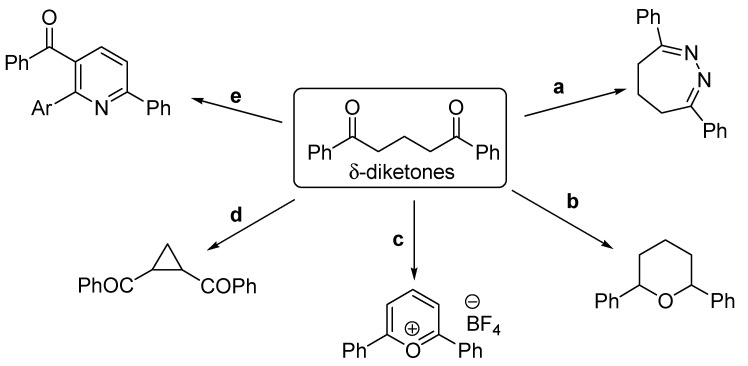
Reactions of δ-diketones leading to various heterocycles and cyclopropanes: **a**—N_2_H_4_, HCl, EtOH, reflux [5]; **b**—InCl_3_ or Al(OTf)_3_, Et_3_SiH,CH_3_CN, r.t. [6]; **c**—BF_3_×Et_2_O, PhMe, 130 °C [7,8]; **d**—NaOH, I_2_, 25–40 °C [9]; **e**—Ni(dppe)Cl_2_, ZnCl_2_, B(OH)_2_Ar, THF, 100 °C [10].

To continue this study, in the current work, we present reactions of 1,5-diarylpent-2-en-4-yn-1-ones with another CH-acid, 3-oxo-3-phenylpropanenitrile (NCCH_2_COPh), that leads to products of Michael addition to the double carbon–carbon bond of starting enynones. The obtained reaction products contain δ-diketones structural fragment. In general, terminally arylated δ-diketones have been widely applied for synthesis of various heterocyclic compounds (Scheme 1) [5,6,7,8,9,10]. Substituted 1,2-diazepines were obtained by interaction of such diketones with hydrazine [5]. These diketones were cyclized into tetrahydropyrans in the reaction with indium chloride (III) and triethylsilane [6]. Cyclization of δ-diketones under the action of boron trifluoride furnished pyrylium salts [7,8]. Reaction of δ-diketones with iodine and sodium hydroxide led to substituted cyclopropanes [9]. There is an interesting example of Ni-catalyzed synthesis of substituted pyridines from 2-benzoyl-5-oxopentanenitriles (δ-dioxonitriles) and arylboronic acids [10]. In this case, the cyano group took part in the pyridine ring formation.

It should be specially emphasized that the above-mentioned heterocycles and cyclopropanes (Scheme 1) have a lot of important practical applications. For instance, 1,2-diazepines exhibit antimicrobial [11], immunomodulatory [12] and antioxidant [13] properties. The tetrahydropyran motif is included in structures of many natural bioactive alkaloids that show toxicity in human tumor cells [14], and have anti-inflammatory properties [15]. A new natural compound, tetrahydropyran containing galiumic acid, reveals antimicrobial activity [16]. Pyrylium salts are widely used as luminescent biolabels for imaging of proteins, nucleic acids, biogenic amines and surface amino groups [17,18,19]. Pyrylium derivatives allow improving mass spectrometry-based detection of peptides [20]. Cyclopropanes are applied as pesticides [21], antiproliferatives [22], drugs for the treatment of osteoporosis [23], and anti-inflammatory agents [24]. 3-Benzoylpyridines show anticonvulsant activity [25]. Apart from that, 3-benzoylpyridines are components of activated delayed fluorescence organic light-emitting diodes [26]. Besides, using 3-benzoylpyridines as an on-tissue cycloaddition reagent for mass spectrometry makes it possible to distinguish a position of the C=C bond in structural isomers of lipids in biological tissues [27].

Thus, a development of methods of synthesis of such δ-diketones and further preparation of various carbo- and hetero-cycles from them are important goals for organic chemistry, biology, medicine, and material science.

## 2. Results and Discussion

Results of reactions of enynones **1a**–**l** with 3-oxo-3-phenylpropanenitrile under the action of bases (sodium methoxide MeONa or LDA) are presented in Table 1. The diastereomeric pairs of polysubstituted δ-diketones **2.1**/**2.2** have been isolated. Structures of the obtained compounds have been determined by means of NMR, IR spectroscopy and HRMS (see Experimental Section and Supplementary Materials). There are two sets of signals in ^1^H- and ^13^C-NMR spectra corresponding to diastereomers **2.1** and **2.2** formed in a constant ratio of 2.5:1. Unfortunately, based on NMR data, we have not been able to identify the exact structure of each stereoisomer.

In most of the cases, the formation of diketones **2.1**/**2.2** takes place at room temperature for 4 h at a ratio of starting enynone **1** and 3-oxo-3-phenylpropanenitrile as 1:1 with MeONa in MeOH (Table 1, entries 1,4–9,12,13). In general, higher yields of the target diketones have been observed for starting enynones **1d**,**e**,**j** bearing electron-withdrawing substituents in aromatic rings Ar^2^ adjacent to the carbonyl group (entries 6,7,12). That is caused by an electron density pulling away from the C=C bond, that makes it more attractive for anion attack. The presence of electron-donating substituents in the ring Ar^2^ at the carbonyl group in enynones **1a**,**c**,**i**,**l** does not significantly decrease the yields of the target diketones **2.1a**,**c**,**i**,**l**/**2.2a**,**c**,**i**,**l** (entries 4,5,11,14). However, it may take a longer reaction time (entries 11,14). On the other hand, enynones **1f**,**g**,**h** bearing electron-donating substituents in aromatic ring Ar^1^ conjugated with acetylene bond give usually lower yields of the corresponding compounds **2.1**/**2.2** (entries 8,9,13). Thus, one may state that the greater influence on electronic structure and reactivity of enynones **1** in these reactions, as well as on the yields of diketones **2.1**/**2.2**, have substituents in aromatic rings Ar^1^ at the triple carbon–carbon bond.

Changing ratio of enynone **1** and 3-oxo-3-phenylpropanenitrile from 1:1 to 2:1 has no effect on the structure of the reaction product (compare entries 1 and 2). It is in contrast with our previous work [4] on the synthesis of multisubstituted cyclohexanes from enynones **1** and malononitrile in the presence of LDA with their ratio of 2:1. The use of LDA as a base in the reaction of enynone **1a** with NCCH_2_COPh results in the same diketones **2.1a**/**2.2a** similarly to the reaction with MeONa (compare entries 1 and 3).

**Scheme 2:**
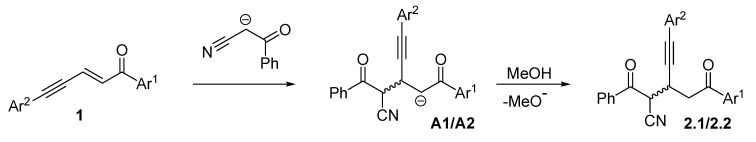
Plausible mechanism of the reaction of enynones **1** with 3-oxo-3-phenylpropanenitrile under basic conditions leading to diastereomeric δ-diketones **2.1**/**2.2**.

A plausible reaction mechanism is given in Scheme 2. The reaction starts from regioselective Michael addition of 3-oxo-3-phenylpropanenitrile anion to the double carbon–carbon bond of starting enynone **1**. At this stage, the anion may attack from both sides of the C=C bond plane, that gives rise to diastereomeric anions **A1**/**A2**. Protonation of the latter affords finally diastereomers of δ-diketones **2.1** and **2.2**.

**Scheme 3:**
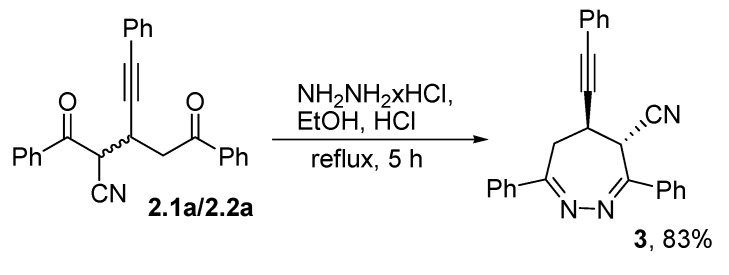
Reaction of δ-diketones **2.1a**/**2.2a** with hydrazine leading to diazepine **3**.

To demonstrate a synthetic potential of the obtained polyfunctional δ-diketones **2.1**/**2.2** we carried out the reaction of compounds **2.1a**/**2.2a** with hydrazine leading to substituted 5,6-dihydro-4*H*-1,2-diazepine **3** solely as a diastereomer in a good yield of 83% (Scheme 3). According to NMR data, there is no NOESY correlation between protons H4 (4.23–4.26 ppm) and H5 (1.26–1.29 ppm) (see Supplementary Materials), that points out *trans*-configuration of substituents at the carbons C4 and C5.

**Scheme 4:**
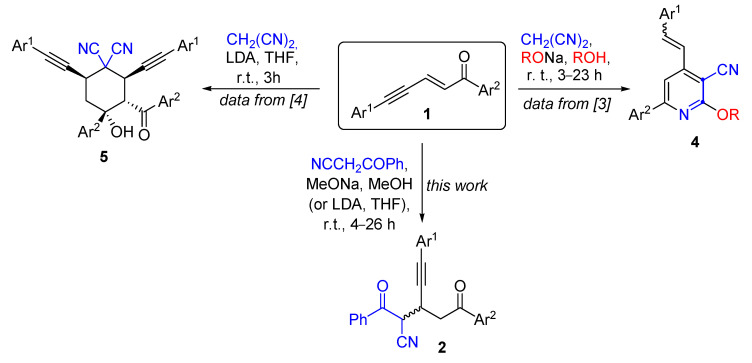
Reactions of enynones **1** with malononitrile [CH_2_(CN)_2_] and 3-oxo-3-phenylpropanenitrile (NCCH_2_COPh) under the basic conditions leading to compounds **2**, **4** and **5**.

Summarizing the date described in this paper and our previous results on reactions of enynones **1** with malononitrile CH_2_(CN)_2_ [3,4], one may state that, depending on the used CH-acid (CH_2_(CN)_2_ or NCCH_2_COPh) and nucleophilicity/basicity of the anionic reagent (RO^−^ or *i-*Pr_2_N^−^), various reaction products may be obtained **2**, **4** or **5** (Scheme 4). The use of strong basic and high nucleophilic alkoxide anions RO^−^ lead to an involvement of this species in the structure of reaction products, pyridines **4** [3]. On the other hand, the use of less nucleophilic anion *i-*Pr_2_N^−^ (due to its large spatial volume), directs the reaction into another pathway with a formation of cyclohexanes **5** [4]. It should be noted that both CH-acids, CH_2_(CN)_2_ and NCCH_2_COPh, give Michael addition product to the C=C bond of substrates **1** at first stages of these reaction. However, this reaction is stopped for NCCH_2_COPh at this step, and goes further for CH_2_(CN)_2_. That is caused by stronger electron acceptor properties of the CN group compared to COPh one. Due to that, there is a possibility of generating of secondary carbanionic species in reactions with CH_2_(CN)_2_. See discussions on the reaction mechanisms in our works [3,4].

## 3. Experimental Section

The NMR spectra of solutions of compounds in CDCl_3_ were recorded at Bruker AVANCE III 400 spectrometer (Karlsruhe, Germany) (at 400 and 100 MHz for ^1^H-, ^13^C-NMR spectra, respectively) at 25 °C. The solvent residual signals of CDCl_3_ (δ 7.26 ppm) for ^1^H-NMR spectra, the carbon signal of CDCl_3_ (δ 77.0 ppm) for ^13^C-NMR spectra were used as references. IR spectra of compounds were taken with Bruker spectrometer (Karlsruhe, Germany). HRMS was carried out at instrument Bruker maXis HRMS-ESI-QTOF (Karlsruhe, Germany). Preparative TLC was performed on silica gel Chemapol L 5/40 (Chemapol, Praga, Zcech Republic).

Preparation and characterization of starting enynones 1 was previously described [28,29].

### 3.1. General Procedure for the Synthesis of 3-(2-Aryl-2-oxoethyl)-5-aryl-2-benzoylpent-4-ynenitriles (***2a**–**l***)

3-oxo-3-phenylpropannitrile (1 equiv.) was added to sodium methoxide (1 equiv.) solution (1.08 mmol/mL) in methanol with stirring. The enynone 1 (1 equiv.) was added to the obtained solution. The reaction mixture was stirred at room temperature for 1–26 h (see Table 1). Then the mixture was poured into water (30 mL), and was extracted with CH_2_Cl_2_ (3 × 30 mL). The combined organic layers were dried with Na_2_SO_4_. Solvent was evaporated under the reduced pressure. A residue was subjected to purification by preparative thin layer chromatography on silica gel using hexane-ethyl acetate mixtures as an eluent. That gave two diastereomers **2.1** and **2.2** in a ratio of 2.5:1.

#### 3.1.1. 2-Benzoyl-3-(2-oxo-2-phenylethyl)-5-phenylpent-4-ynenitrile (**2.1a**/**2.2a**)

Mixture of diastereomers. Obtained from **1a** (50 mg, 0.22 mmol) in a yield of 75 mg (90%). Obtained from **1a** (50 mg, 0.22 mmol) and 3-oxo-3-phenylpropanenitrile 79.8 mg (0.55 mmol) in a yield of 70 mg (84%). Obtained from **1a** (50 mg, 0.22 mmol) and 3-oxo-3-phenylpropanenitrile (31.0 mg, 0.22 mmol) in presence of LDA (0.22 mmol) in a yield of 66 mg (79%). Oilish compound. ^1^H-NMR, **2.1a** (selected signals of major isomer, obtained from spectrum of mixture of stereoisomers) δ, ppm: 3.81 dd (1H, CH_2_, *J* = 9.9, 18.7 Hz), 5.20 (1H, CH-CN, *J* = 4.3 Hz), 8.08 t (2H, Ph, *J* = 7.2 Hz), 8.23 d (2H, Ph, *J* = *7*.4 Hz). ^1^H-NMR, **2.2a** (selected signals of minor isomer, obtained from spectrum of mixture of stereoisomers) δ, ppm: 5.13 d (1H, CH-CN, *J* = 6.3 Hz), 8.02 d (2H, Ph, *J* = 7.3 Hz). ^1^H-NMR, (signals of mixture of isomers) δ, ppm: 3.61–3.35 m (3H, CH_2_), 4.09–4.15 m (2H, CH-CC), 7.23–7.26, 7.30–7.33, 7.41–7.43, 7.49–7.72 m. ^13^C-NMR, **2.1a** (selected signals of major isomer, obtained from spectrum of mixture of stereoisomers) δ, ppm: 29.3, 41.7, 45.7, 84.87 (C≡), 86.4 (C≡), 115.5 (CN), 122.18, 189.4 (CO), 197.3 (CO). ^13^C-NMR, **2.2a** (selected signals of minor isomer, obtained from spectrum of mixture of stereoisomers) δ, ppm: 29.0, 40.6, 43.5, 84.89 (C≡), 86.7 (C≡), 115.8 (CN), 122.15, 189.5 (CO), 196.2 (CO). ^13^C-NMR, (signals of mixture of isomers) δ, ppm: 128.14, 125.22, 128.23, 128.47, 128.58, 128.8, 128.9, 129.0, 129.1, 129.2, 131.7, 131.9, 133.7, 133.95, 134.14, 134.38, 134.43, 134.8, 135.9, 136.4. IR (KBr): 1684 cm^−1^ (CO), 2249 cm^−1^ (CN, C≡C). HRMS (ESI) [M + Na]^+^: Calcd for C_26_H_19_NO_2_Na 400.1313, Found 400.1308.

#### 3.1.2. 2-Benzoyl-3-(2-(4-methylphenyl)-2-oxo-ethyl)-5-phenylpent-4-ynenitrile (**2.1b**/**2.2b**)

Mixture of diastereomers. Obtained from **1b** (50 mg, 0.20 mmol) in a yield of 72 mg (92%). Oilish compound. ^1^H-NMR, **2.1b** (selected signals of major isomer, obtained from spectrum of mixture of stereoisomers) δ, ppm: 2.47 s (3H, CH_3_), 3.78 dd (1H, CH_2_, *J* = 10.0, 18.7 Hz), 5.20 d (1H, CH-CN, *J* = 4.3 Hz), 7.97 d (2H, Ar, *J* = 8.2 Hz), 8.24 d (2H, Ar, *J* = 7.2 Hz). ^1^H-NMR, **2.2b** (selected signals of minor isomer, obtained from spectrum of mixture of stereoisomers) δ, ppm: 2.44 s (3H, CH_3_), 5.14 d (1H, CH-CN, *J* = 6.4 Hz), 7.92 d (2H, Ar, *J* = 8.3 Hz), 8.09 d (2H, Ar, *J* = 7.2 Hz). ^1^H-NMR, (signals of mixture of isomers) δ, ppm: 3.57–3.62 m (3H, CH_2_), 4.07–4.14 m (2H, CH-CC), 7.22–7.35, 7.41–7.44, 7.54–7.62, 7.67–7.71 m. ^13^C-NMR, **2.1b** (selected signals of major isomer, obtained from spectrum of mixture of stereoisomers) δ, ppm: 21.8 (CH_3_), 29.4, 41.6, 45.8, 85.0 (C≡), 86.4 (C≡), 115.6 (CN), 122.23, 145.2, 189.5 (CO), 196.9 (CO). ^13^C-NMR, **2.2b** (selected signals of minor isomer, obtained from spectrum of mixture of stereoisomers) δ, ppm: 21.8 (CH_3_), 29.4, 41.6, 45.8, 85.0 (C≡), 86.4 (C≡), 115.6 (CN), 122.23, 145.2, 189.5 (CO), 196.9 (CO). ^13^C-NMR, (signals of mixture of isomers) δ, ppm:128.1, 128.2, 128.35, 128.44, 128.48, 128.55, 129.0, 129.1, 129.2, 129.4, 129.6, 131.7, 131.9, 133.4, 133.94, 133.97, 134.4, 134.7. IR (KBr): 1681 cm^−1^ (CO), 2250 cm^−1^ (CN, C≡C). HRMS (ESI) [M + Na]^+^: Calcd for C_27_H_21_NO_2_Na 414.1470, Found 414.1465.

#### 3.1.3. 2-Benzoyl-3-(2-(4-methoxyphenyl)-2-oxo-ethyl)-5-phenylpent-4-ynenitrile (**2.1c**/**2.2c**)

Mixture of diastereomers. Obtained from **1c** (50 mg, 0.19 mmol) in a yield of 72 mg (94%). Oilish compound. ^1^H-NMR, **2.1c** (selected signals of major isomer, obtained from spectrum of mixture of stereoisomers) δ, ppm: 3.92 s (3H, OCH_3_), 3.76 dd (1H, CH_2_, *J* = 10.1, 18.5 Hz),5.21 d (1H, CH-CN, *J* = 4.2 Hz), 7.01 d (2H, Ar, *J* = 8.9 Hz), 8.05 d (2H, Ar, *J* = 8.9 Hz), 8.24 d (2H, Ar, *J* = 7.2 Hz). ^1^H-NMR, **2.2c** (selected signals of minor isomer, obtained from spectrum of mixture of stereoisomers) δ, ppm: 3.90 s (3H, OCH_3_), 5.14 d (1H, CH-CN, *J* = 6.3 Hz), 6.98 d (2H, Ar, *J* = 8.9 Hz), 8.00 d (2H, Ar, *J* = 8.9 Hz), 8.08 d (2H, Ar, *J* = 7.3 Hz). ^1^H-NMR, (signals of mixture of isomers) δ, ppm: 3.53–3.59 m (3H, CH_2_), 3.90–4.13 m (2H, CH-CC), 7.22–7.33, 7.41–7.43, 7.54–7.62, 7.61–7.71 m. ^13^C-NMR, **2.1c** (selected signals of major isomer, obtained from spectrum of mixture of stereoisomers) δ, ppm: 29.5, 41.3, 45.9, 55.62 (OCH_3_), 85.0 (C≡), 86.4 (C≡), 114.1, 115.6 (CN), 122.23, 130.61, 131.9, 133.9, 134.41, 164.3 (C-O, Ar), 189.57 (CO), 195.7 (CO). ^13^C-NMR, **2.2c** (selected signals of minor isomer, obtained from spectrum of mixture of stereoisomers) δ, ppm: 29.1, 40.1, 43.6, 55.56 (OCH_3_), 84.8 (C≡), 86.9 (C≡), 113.9, 115.9 (CN), 122.22, 130.56, 131.7, 134.39, 134.7, 164.0 (C-O, Ar), 189.62 (CO), 194.6 (CO). ^13^C-NMR, (signals of mixture of isomers) δ, ppm: 128.1, 128.2, 128.4, 1528.5, 128.6, 128.9, 129.06, 129.09, 129.2, 129.5. IR (KBr): 1673 cm^−1^, 1689 cm^−1^ (CO), 2250 cm^−1^ (CN, C≡C). HRMS (ESI) [M + Na]^+^: Calcd for C_27_H_21_NO_3_Na 430.1419, Found 430.1414.

#### 3.1.4. 2-Benzoyl-3-(2-(4-fluorophenyl)-2-oxoethyl)-5-phenylpent-4-ynenitrile (**2.1d**/**2.2d**)

Mixture of diastereomers. Obtained from **1d** (30 mg, 0.12 mmol) in a yield of 46 mg (98%). Oilish compound. ^1^H-NMR, **2.1d** (selected signals of major isomer, obtained from spectrum of mixture of stereoisomers) δ, ppm: 3.76 dd (1H, CH_2_, *J* = 9.7, 18.6 Hz), 5.18 d (1H, CH-CN, *J* = 4.4 Hz), 7.69 t (1H, Ar, *J* = 7.4 Hz), 8.22 (2H, Ar, *J* = 7.3 Hz). ^1^H-NMR, **2.2d** (selected signals of minor isomer, obtained from spectrum of mixture of stereoisomers) δ, ppm: 5.11 d (1H, CH-CN, *J* = 6.1 Hz). ^1^H-NMR, (signals of mixture of isomers) δ, ppm: 3.57–3.62 m (3H, CH_2_), 4.08–4.14 m (2H, CH-CC), 7.13–7.25 m, 7.30–7.33 m, 7.40–7.42 m, 7.55–7.62 m, 8.04–8.12 m. ^13^C-NMR, **2.1d** (selected signals of major isomer, obtained from spectrum of mixture of stereoisomers) δ, ppm: 29.3, 41.6, 45.6, 84.8 (C≡), 86.5 (C≡), 115.5 (CN), 116.2 d (Ar, *p*-C-F, *J* = 22.0 Hz), 122.12, 189.4 (CO), 195.7 (CO). ^13^C-NMR, **2.2d** (selected signals of minor isomer, obtained from spectrum of mixture of stereoisomers) δ, ppm: 28.9, 40.5, 43.6, 84.9 (C≡), 86.7 (C≡), 115.76 (CN), 115.9 d (Ar, *p*-C-F, *J* = 22.0 Hz), 122.10, 189.5 (CO), 194.6 (CO). ^13^CNMR, (signals of mixture of isomers) δ, ppm:128.2, 128.3, 128.55, 128.56, 128.63, 129.0, 129.1, 129.2, 130.9, 130.96, 131.03, 131.7, 131.9, 132.1, 133.9, 134.3, 134.5, 134.8, 165.2 d (Ar, *i*-C-F, *J* = 32.9 Hz), 167.2 d (Ar, *i*-C-F, *J* = 33.8 Hz). IR (KBr): 1674 cm^−1^ (CO), 1690 cm^−1^ (CO), 2249 cm^−1^ (CN, C≡C). HRMS (ESI) [M + Na]^+^: Calcd for C_26_H_18_NO_2_FNa 419.1219, Found 418.1214.

#### 3.1.5. 2-Benzoyl-3-(2-(4-chlorophenyl)-2-oxoethyl)-5-phenylpent-4-ynenitrile (**2.1e**/**2.2e**)

Mixture of diastereomers. Obtained from **1e** (30 mg, 0.11 mmol) in a yield of 43 mg (96%). Oilish compound. ^1^H-NMR, **2.1e** (selected signals of major isomer, obtained from spectrum of mixture of stereoisomers) δ, ppm: 3.75 dd (1H, CH_2_, *J* = 9.6, 18.7 Hz), 5.16 d (1H, CH-CN, *J* = 4.4 Hz), 7.41 dd (2H, Ar, *J* = 1.9, 7.6 Hz), 7.52 d (2H, Ar, *J* = 8.5 Hz), 7.61 d (2H, Ar, *J* = 7.9 Hz), 7.70 t (1H, Ar, *J* = 7.4 Hz), 8.01 d (2H, Ar, *J* = 8.5 Hz), 8.21 (2H, Ar, *J* = 7.2 Hz). ^1^H-NMR, **2.2e** (selected signals of minor isomer, obtained from spectrum of mixture of stereoisomers) δ, ppm: 5.09 d (1H, CH-CN, *J* = 6.1 Hz), 7.49 d (2H, Ar, *J* = 8.5 Hz), 7.96 d (2H, Ar, *J* = 8.6 Hz), 8.08 d (2H, Ar, 7.3 Hz). ^1^H-NMR, (signals of mixture of isomers) δ, ppm: 3.57–3.62 m (3H, CH_2_), 4.08–4.14 m (2H, CH-CC), 7.24–7.33, 7.57–7.59 m. ^13^C-NMR, **2.1e** (selected signals of major isomer, obtained from spectrum of mixture of stereoisomers) δ, ppm: 29.3, 41.7, 45.6, 84.7 (C≡), 86.5 (C≡), 115.5 (CN), 122.08, 140.7 (Ar, C-Cl), 189.3 (CO), 196.1 (CO). ^13^C-NMR, **2.2e** (selected signals of minor isomer, obtained from spectrum of mixture of stereoisomers) δ, ppm: 28.8, 40.6, 43.6, 85.0 (C≡), 86.6 (C≡), 115.7 (CN), 122.05, 140.2 (Ar, C-Cl), 189.4 (CO), 195.0 (CO). ^13^C-NMR, (signals of mixture of isomers) δ, ppm: 128.19, 128.24, 128.56, 128.64, 129.0, 129.07, 129.11, 129.2, 129.3, 129.6, 131.7, 131.9, 133.9, 134.2, 134.3, 134.5, 134.7, 134.8. IR (KBr): 1689 cm^−1^ (CO), 2252 cm^−1^ (CN, C≡C). HRMS (ESI) [M + Na]^+^: Calcd for C_26_H_18_NO_2_ClNa 434.0924, Found 434.0918.

#### 3.1.6. 2-Benzoyl-3-(2-oxo-2-phenylethyl)-5-(p-tolyl)pent-4-ynenitrile (**2.1f**/**2.2f**)

Mixture of diastereomers. Obtained from **1f** (50 mg, 0.20 mmol) in a yield of 61 mg (78%). Oilish compound. ^1^H-NMR, **2.1f** (selected signals of major isomer, obtained from spectrum of mixture of stereoisomers) δ, ppm: 2.35 s (3H, CH_3_), 3.79 dd (1H, CH_2_, *J* = 9.8, 18.7 Hz), 5.19 d (1H, CH-CN, *J* = 4.3 Hz), 7.31 d (2H, Ar, *J* = 8.0 Hz), 8.23 d (2H, Ar, *J* = 7.4 Hz). ^1^H-NMR, **2.2f** (selected signals of minor isomer, obtained from spectrum of mixture of stereoisomers) δ, ppm: 2.32 s (3H, CH_3_), 5.12 d (1H, CH-CN, *J* = 6.3 Hz), 7.04 d (2H, Ar, *J* = 7.8 Hz), 8.02 d (2H, Ar, *J* = 7.4 Hz).). ^1^H-NMR, (signals of mixture of isomers) δ, ppm: 3.59–3.64 m (3H, CH_2_), 4.08–4.14 m (2H, CH-CC), 7.10–7.14 m, 7.51–7.69 m, 8.06–8.09 m. ^13^C-NMR, **2.1f** (selected signals of major isomer, obtained from spectrum of mixture of stereoisomers) δ, ppm: 21.5 (CH_3_), 29.4, 41.8, 45.8, 84.2 (C≡), 86.6 (C≡), 115.6 (CN), 119.12, 135.9, 138.7, 189.5 (CO), 197.4 (CO). ^13^C-NMR, **2.2f** (selected signals of minor isomer, obtained from spectrum of mixture of stereoisomers) δ, ppm: 21.4 (CH_3_), 29.0, 40.7, 43.7, 85.1 (C≡), 86.0 (C≡), 115.9 (CN), 119.08, 136.4, 138.6, 189.6 (CO), 196.2 (CO). ^13^C-NMR, (signals of mixture of isomers) δ, ppm: 128.2, 128.8, 128.91, 128.93, 128.98, 129.03, 129.1, 129.2, 131.6, 131.8, 133.6, 134.0, 134.1, 134.4, 134.7. IR (KBr): 1686 cm^−1^ (CO), 2252 cm^−1^ (CN, C≡C). HRMS (ESI) [M + Na]^+^: Calcd for C_27_H_21_NO_2_Na 414.1470, Found 414.1465. 

#### 3.1.7. 2-Benzoyl-5-(4-methoxyphenyl)-3-(2-oxo-2-phenylethyl)pent-4-ynenitrile (**2.1g**/**2.2g**)

Mixture of diastereomers. Obtained from **1g** (30 mg, 0.11 mmol) in a yield of 24 mg (53%). Oilish compound. ^1^H-NMR, **2.1g** (selected signals of major isomer, obtained from spectrum of mixture of stereoisomers) δ, ppm: 3.82 s (3H, OCH_3_), 5.19 d (1H, CH-CN, *J* = 4.3 Hz), 6.83 d (2H, Ar, *J* = 8.8 Hz), 7.34 d (2H, Ar, *J* = 8.8 Hz), 7.95 d (2H, Ar, *J* = 7.8 Hz), 8.23 d (2H, Ar, *J* = 7.3 Hz). ^1^H-NMR, **2.2g** (selected signals of minor isomer, obtained from spectrum of mixture of stereoisomers) δ, ppm: 3.79 s (3H, OCH_3_), 5.11 d (1H, CH-CN, *J* = 6.3 Hz), 6.76 d (2H, Ar, *J* = 8.8 Hz), 7.16 d (2H, Ar, *J* = 8.8 Hz), 8.02 d (2H, Ar, *J* = 7.5 Hz). ^1^H-NMR, (signals of mixture of isomers) δ, ppm: 3.58–3.64 m (3H, CH_2_), 3.75–3.84 m (1H, CH_2_), 4.07–4.10 m (2H, CH-CC), 7.51–7.69 m, 8.06–8.09 m. ^13^C-NMR, **2.1g** (selected signals of major isomer, obtained from spectrum of mixture of stereoisomers) δ, ppm: 29.4, 41.9, 45.9, 55.30 (OCH_3_), 85.4 (C≡), 86.4 (C≡), 115.6 (CN), 159.8 (Ar, C-O), 189.5 (CO), 197.4 (CO). ^13^C-NMR, **2.2g** (selected signals of minor isomer, obtained from spectrum of mixture of stereoisomers) δ, ppm: 29.1, 40.7, 43.7, 55.25 (OCH_3_), 84.9 (C≡), 85.3 (C≡), 115.9 (CN), 159.7 (Ar, C-O), 189.7 (CO), 196.3 (CO). ^13^C-NMR, (signals of mixture of isomers) δ, ppm: 113.8, 113.9, 114.2, 114.3, 128.2, 128.5, 128.8, 128.9, 129.0, 129.1, 129.2, 133.1, 133.3, 133.6, 134.0, 134.1, 134.3, 134.4, 134.7, 135.9, 136.4. IR (KBr): 1691 cm^−1^ (CO), 2252 cm^−1^ (CN, C≡C). HRMS (ESI) [M + Na]^+^: Calcd fo rC_27_H_21_NO_3_Na 430.1419, Found 430.1414.

#### 3.1.8. 2-Benzoyl-5-(4-chlorophenyl)-3-(2-oxo-2-phenylethyl)pent-4-ynenitrile (**2.1h**/**2.2h**)

Mixture of diastereomers. Obtained from **1h** (30 mg, 0.11 mmol) in a yield of 37 mg (82%). Oilish compound. ^1^H-NMR, **2.1h** (selected signals of major isomer, obtained from spectrum of mixture of stereoisomers) δ, ppm: 3.79 dd (1H, CH_2_, *J* = 9.8, 18.7 Hz), 5.18 d (1H, CH-CN, *J* = 4.3 Hz), 7.28 d (2H, Ar, *J* = 8.4 Hz), 7.34 d (2H, Ar, *J* = 8.6 Hz), 8.23 d (2H, Ar, *J* = 7.2 Hz). ^1^H-NMR, **2.2h** (selected signals of minor isomer, obtained from spectrum of mixture of stereoisomers) δ, ppm: 5.12 d (1H, CH-CN, *J* = 6.4 Hz), 7.15 d (2H, Ar, *J* = 8.6 Hz), 7.21 d (2H, Ar, *J* = 8.5 Hz), 8.02 d (2H, Ar, *J* = 7.2 Hz). ^1^H-NMR, (signals of mixture of isomers) δ, ppm: 3.59–3.64 m (3H, CH_2_), 4.08–4.13 m (2H, CH-CC), 7.50–7.72 m, 8.06–8.09 m. ^13^C-NMR, **2.1h** (selected signals of major isomer, obtained from spectrum of mixture of stereoisomers) δ, ppm: 29.3, 41.6, 45.6, 85.3 (C≡), 86.0 (C≡), 115.5 (CN), 120.66, 135.8, 189.3 (CO), 197.2 (CO). ^13^C-NMR, **2.2h** (selected signals of minor isomer, obtained from spectrum of mixture of stereoisomers) δ, ppm: 28.9, 40.5, 43.3, 83.8 (C≡), 87.8 (C≡), 115.7 (CN), 120.64, 136.3, 189.4 (CO), 196.1 (CO). ^13^C-NMR, (signals of mixture of isomers) δ, ppm: 128.20, 128.23, 128.5, 128.6, 128.8, 128.96, 129.04, 129.1, 129.21, 129.23, 132.9, 133.1, 133.7, 133.8, 134.2, 134.3, 134.6, 134.7, 134.8. IR (KBr): 1685 cm^−1^ (CO), 2250 cm^−1^ (CN, C≡C). HRMS (ESI) [M + Na]^+^: Calcd for C_26_H_18_NO_2_ClNa 434.0924, Found 434.0918.

#### 3.1.9. 2-Benzoyl-3-(2-(3,4-dimethoxyphenyl)-2-oxoethyl)-5-phenylpent-4-ynenitrile (**2.1i**/**2.2i**)

Mixture of diastereomers. Obtained from **1i** (30 mg, 0.10 mmol) in a yield of 27 mg (63%). Oilish compound. ^1^H-NMR, **2.1i** (selected signals of major isomer, obtained from spectrum of mixture of stereoisomers) δ, ppm: 3.77 dd (1H, CH_2_, *J* = 10.1, 18.5 Hz), 4.00 s (6H, OCH_3_), 5.19 d (1H, CH-CN, *J* = 4.2 Hz), 6.96 d (2H, Ar, *J* = 8.5 Hz), 8.23 d (2H, Ar, *J* = 7.4 Hz). ^1^H-NMR, **2.2i** (selected signals of minor isomer, obtained from spectrum of mixture of stereoisomers) δ, ppm: 3.96 s (3H, OCH_3_), 3.98 s (3H, OCH_3_), 5.14 d (1H, CH-CN, *J* = 6.4 Hz), 6.93 d (2H, Ar, *J* = 8.4 Hz), 7.94 d (2H, Ar, *J* = 8.1 Hz), 8.09 d (2H, Ar, *J* = 7.6 Hz). ^1^H-NMR, (signals of mixture of isomers) δ, ppm: 3.54–3.59 m (3H, CH_2_), 4.08–4.13 m (2H, CH-CC), 7.22–7.23, 7.30–7.33, 7.41–7.43, 7.54–7.7.62, 7.65–7.73 m. ^13^C-NMR, **2.1i** (selected signals of major isomer, obtained from spectrum of mixture of stereoisomers) δ, ppm: 29.5, 41.2, 45.9, 56.1 (OCH_3_), 56.21 (OCH_3_), 85.0 (C≡), 86.4 (C≡),110.0, 110.3, 115.6 (CN), 149.3, 154.2, 189.5 (CO), 195.7 (CO). ^13^C-NMR, **2.2i** (selected signals of minor isomer, obtained from spectrum of mixture of stereoisomers) δ, ppm: 29.2, 40.0, 43.6, 56.0 (OCH_3_), 56.15 (OCH_3_), 84.9 (C≡), 86.9 (C≡), 110.1, 110.2, 115.9 (CN), 149.2, 153.8, 189.6 (CO), 194.7 (CO). ^13^C-NMR, (signals of mixture of isomers) δ, ppm: 122.2, 123.1, 123.3, 128.1, 128.2, 128.45, 125.47, 128.6, 129.0, 129.07, 129.08, 129.2, 129.6, 131.7, 131.9, 132.1, 132.2, 133.9, 134.4, 134.72, 134.74. IR (KBr): 1695 cm^−1^ (CO), 2252 cm^−1^ (CN, C≡C). HRMS (ESI) [M + Na]^+^: Calcd for C_28_H_23_NO_4_Na 460.1525, Found 460.1519.

#### 3.1.10. 2-Benzoyl-3-(2-(4-chlorophenyl)-2-oxoethyl)-5-(*p*-tolyl)pent-4-ynenitrile (**2.1j**/**2.2j**)

Mixture of diastereomers. Obtained from **1j** (50 mg, 0.18 mmol) in a yield of 75 mg (97%). Oilish compound. ^1^H-NMR, **2.1j** (selected signals of major isomer, obtained from spectrum of mixture of stereoisomers) δ, ppm: 2.36 s (3H, CH_3_), 3.74 dd (1H, CH_2_, *J* = 9.6, 18.6 Hz), 5.15 d (1H, CH-CN, *J* = 4.4 Hz), 7.29 d (2H, Ar, *J* = 8.0 Hz), 7.52 d (2H, Ar, *J* = 8.6 Hz), 8.01 d (2H, Ar, *J* = 8.6 Hz), 8.21 d (2H, Ar, *J* = 7.4 Hz). ^1^H-NMR, **2.2j** (selected signals of minor isomer, obtained from spectrum of mixture of stereoisomers) δ, ppm: 2.32 s (3H, CH_3_), 5.08 d (1H, CH-CN, *J* = 6.1 Hz), 7.05 d (2H, Ar, *J* = 8.0 Hz), 7.48 d (2H, Ar, *J* = 8.6 Hz), 8.57 d (2H, Ar, *J* = 8.6 Hz), 8.07 d (2H, Ar, *J* = 7.5 Hz). ^1^H-NMR, (signals of mixture of isomers) δ, ppm: 3.55–3.61 m (3H, CH_2_), 4.07–4.11 m (2H, CH-CC), 7.10–7.14, 7.57–7.61, 7.68–7.71 m. ^13^C-NMR, **2.1j** (selected signals of major isomer, obtained from spectrum of mixture of stereoisomers) δ, ppm: 21.51 (CH_3_), 29.3, 41.8, 45.7, 83.9 (C≡), 86.7 (C≡), 115.5 (CN), 118.99, 138.8, 140.7, 189.4 (CO), 196.2 (CO). ^13^C-NMR, **2.2j** (selected signals of minor isomer, obtained from spectrum of mixture of stereoisomers) δ, ppm: 21.46 (CH_3_), 29.9, 40.7, 43.7, 85.1 (C≡), 85.8 (C≡), 115.8 (CN), 118.95, 138.7, 140.2, 189.5 (CO), 195.1 (CO). ^13^C-NMR, (signals of mixture of isomers) δ, ppm: 128.5, 128.9, 128.99, 129.02, 129.08, 129.10, 129.17, 129.20, 129.3, 129.6, 131.6, 131.8, 133.9, 134.2, 134.5, 134.7, 134.75, 134.82. IR (KBr): 1687 cm^−1^ (CO), 2250 cm^−1^ (CN, C≡C). HRMS (ESI) [M + Na]^+^: Calcd for C_27_H_20_ClNO_2_Na 448.1080, Found 448.1075.

#### 3.1.11. 2-Benzoyl-3-(2-(4-bromphenyl)-2-oxoethyl)-5-(*p*-tolyl)pent-4-ynenitrile (**2.1k**/**2.2k**)

Mixture of diastereomers. Obtained from **1k** (50 mg, 0.15 mmol) in a yield of 54 mg (77%). Oilish compound. ^1^H-NMR, **2.1k** (selected signals of major isomer, obtained from spectrum of mixture of stereoisomers) δ, ppm: 2.35 s (3H, CH_3_), 3.73 dd (1H, CH_2_, *J* = 9.6, 18.6 Hz), 5.15 d (1H, CH-CN, *J* = 4.4 Hz), 7.29 d (2H, Ar, *J* = 8.0 Hz), 7.92 d (2H, Ar, *J* = 8.5 Hz), 8.20 d (2H, Ar, *J* = 7.4 Hz). ^1^H-NMR, **2.2k** (selected signals of minor isomer, obtained from spectrum of mixture of stereoisomers) δ, ppm: 2.32 s (3H, CH_3_), 5.08 d (1H, CH-CN, *J* 6.1 Hz), 7.05 d (2H, Ar, *J* = 8.0 Hz), 7.87 d (2H, Ar, *J* = 8.5 Hz), 8.07 d (2H, Ar, *J* = 7.4 Hz). ^1^H-NMR, (signals of mixture of isomers) δ, ppm:3.54–3.60 m (3H, CH_2_), 4.06–4.12 m (2H, CH-CC), 7.09–7.14 m, 7.56–7.69 m. ^13^C-NMR, **2.1k** (selected signals of major isomer, obtained from spectrum of mixture of stereoisomers) δ, ppm: 21.49 (CH_3_), 29.3, 41.7, 45.7, 84.0 (C≡), 86.7 (C≡), 115.5 (CN), 118.96, 131.8, 132.3, 134.0, 134.5, 134.6, 138.8, 189.4 (CO), 196.4 (CO). ^13^C-NMR, **2.2j** (selected signals of minor isomer, obtained from spectrum of mixture of stereoisomers) δ, ppm: 21.45 (CH_3_), 28.8, 40.6, 43.7, 85.2 (C≡), 85.8 (C≡), 115.8 (CN), 119.00, 131.6, 132.1, 134.3, 134.8, 135.1, 138.7, 189.5 (CO), 195.3 (CO). ^13^C-NMR, (signals of mixture of isomers) δ, ppm:128.91, 128.94, 128.99, 129.00, 129.1, 129.2, 129.5, 129.70, 129.73. IR (KBr): 1687 cm^−1^ (CO), 2250 cm^−1^ (CN, C≡C). HRMS (ESI) [M + Na]^+^: Calcd for C_27_H_20_BrNO_2_Na 492.0575, Found 492.0570.

#### 3.1.12. 2-Benzoyl-3-(2-oxo-2-(thiophen-2-yl)ethyl)-5-(*p*-tolyl)pent-4-ynenitrile (**2.1l**/**2.2l**)

Mixture of diastereomers. Obtained from **1l** (30 mg, 0.12 mmol) in a yield of 46 mg (96%). Oilish compound. ^1^H-NMR, **2.1l** (selected signals of major isomer, obtained from spectrum of mixture of stereoisomers) δ, ppm: 2.35 s (3H, CH_3_), 3.73 dd (1H, CH_2_, *J* = 9.9, 18.2 Hz), 5.17 d (1H, CH-CN, *J* = 4.4 Hz), 7.30 d (2H, Ar, *J* = 8.1 Hz), 7.76 d (1H, thio, *J* = 4.9 Hz), 7.88 d (1H, thio, *J* = 3.8 Hz), 8.19 d (2H, Ar, *J* = 7.5 Hz). ^1^H-NMR, **2.2l** (selected signals of minor isomer, obtained from spectrum of mixture of stereoisomers) δ, ppm: 2.31 s (3H, CH_3_), 5.13 d (1H, CH-CN, *J* = 6.5 Hz), 7.83 d (1H, thio, *J* = 3.4 Hz), 8.08 d (2H, Ar, *J* = 7.6 Hz). ^1^H-NMR, (signals of mixture of isomers) δ, ppm: 3.52–3.58 m (3H, CH_2_), 4.05–4.10 m (2H, CH-CC), 7.02–7.12, 7.19–7.23, 7.54–7.60, 7.67–7.71 m. ^13^C-NMR, **2.1l** (selected signals of major isomer, obtained from spectrum of mixture of stereoisomers) δ, ppm: 21.5 (CH_3_), 29.5, 41.0, 45.8, 83.9 (C≡), 86.7 (C≡), 115.5 (CN), 119.04, 138.8, 143.0, 189.4 (CO), 190.1 (CO). ^13^C-NMR, **2.2l** (selected signals of minor isomer, obtained from spectrum of mixture of stereoisomers) δ, ppm: 21.4 (CH_3_), 29.3, 41.0, 43.5, 85.4 (C≡), 85.6 (C≡), 115.8 (CN), 119.00, 138.6, 143.6, 189.1 (CO), 189.6 (CO). ^13^C-NMR, (signals of mixture of isomers) δ, ppm: 128.3, 128.6, 128.88, 128.97, 129.01, 129.16, 129.18, 131.6, 131.8, 132.7, 133.2, 133.9, 134.4, 134.5, 134.7, 135.0. IR (KBr): 1660 cm^−1^ (CO), 1693 cm^−1^ (CO), 2250 cm^−1^ (CN, C≡C). HRMS (ESI) [M + Na]^+^: Calcd for C_25_H_19_NO_2_SNa 420.1034, Found 420.1029.

### 3.2. Procedure for the Synthesis of 5,6-Dihydro-4H-1,2-diazepine (**3**)

A total of 0.23 mL of hydrazine hydrochloride (3.4 mg, 0.8 mmol) solution (15 mg/mL) in ethanol was added to mixture of diastereomers **2.1a**/**2.2a** (30 mg, 0.8 mmol). The mixture was diluted with 2 mL of ethanol. Three drops of concentrated hydrochloric acid were added. The mixture was refluxed for 5 h. The reaction was monitored by TLC. Then, the mixture was poured into water (30 mL), and was extracted with CH_2_Cl_2_ (3 × 30 mL). The combined organic layers were washed with water (30 mL) and were dried with Na_2_SO_4_. Solvent was evaporated under the reduced pressure, that gave compound **3** in the yield of 25 mg (83%).

#### 4,5-*trans*-4-Cyano-3,7-diphenyl-5-(phenylethynyl)-5,6-dihydro-4*H*-1,2-diazepine (**3**)

Oilish compound. ^1^H-NMR, ppm: 1.26–1.29 m (1H, CH-C≡), 3.39 dd (1H, CH_2_, *J =* 2.16, 13.5 Hz), 3.63 dd (1H, CH_2_, *J* = 6.7, 13.5 Hz), 4.24 dd (1H, CH-CN, *J* = 2.1, 6.6 Hz), 7.19–7.27 m (5H, Ph), 7.41–7.46 m (3H, Ph), 7.50–7.51 m (3H, Ph), 7.63–7.66 m (2H, Ph), 7.84–7.88 m (2H, Ph). ^13^C-NMR, ppm: 29.7 (CH-C≡), 33.9 (CH_2_), 36.1 (CH-CN), 83.7 (C≡), 85.3 (C≡), 119.1 (C≡N), 120.7, 122.6, 126.7, 128.1, 128.6, 128.7, 129.1, 129.8, 130.7, 131.7, 134.0, 137.7, 153.2 (C=N), 128.8 (C=N). HRMS (ESI) [M + Na]^+^: Calcd for C_26_H_19_N_3_Na 396.1477, Found 396.1471.

## 4. Conclusions

We have developed a synthesis of novel polyfunctional δ-diketones by Michael addition of 3-oxo-3-phenylpropanenitrile to conjugated 1,5-diarylpent-1-en-4-yn-1-ones under the action of sodium methoxide in methanol. The obtained δ-diketones are promising precursors for synthesis of various heterocycles.

## Figures and Tables

**Table 1:**
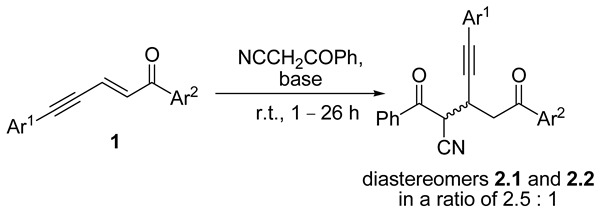
Reactions of enynones **1** with 3-oxo-3-phenylpropanenitrile and sodium methoxide in methanol leading to δ-diketones **2.1**/**2.2**.

Entry	Starting Enynone	Reaction Conditions: Base–Solvent, Temperature, Time, R—Ratio of Enynone 1 and NCCH_2_COPh	Reaction Products, Ratio of Diastereomers, Yield
1	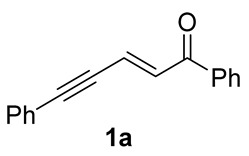	MeONa, MeOH, r.t., 4 h, R = 1:1	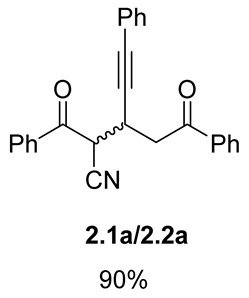
2	**1a**	MeONa, MeOH, r.t., 3 h, R = 2:1	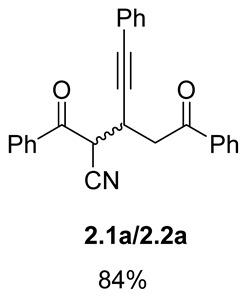
3	**1a**	LDA, THF, r.t., 1 h, R = 2:1	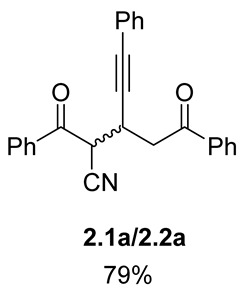
4	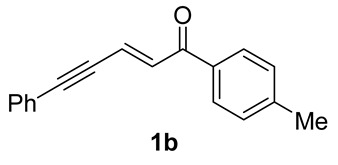	MeONa, MeOH, r.t., 4 h, R = 1:1	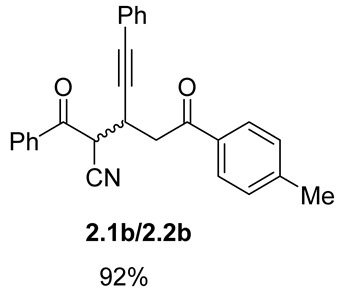
5	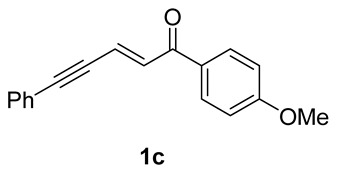	MeONa, MeOH, r.t., 4 h, R = 1:1	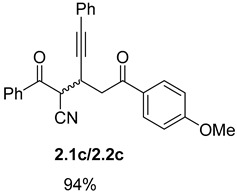
6	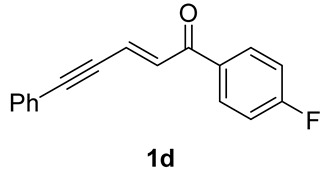	MeONa, MeOH, r.t., 5 h, R = 1:1	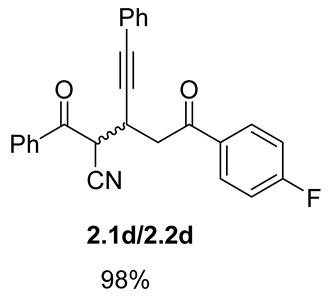
7	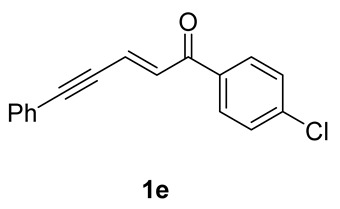	MeONa, MeOH, r.t., 4 h, R = 1:1	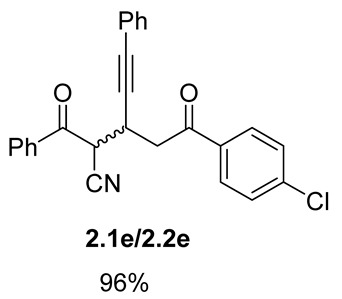
8	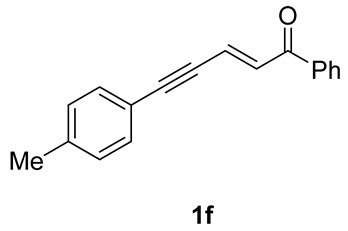	MeONa, MeOH, r.t., 4 h, R = 1:1	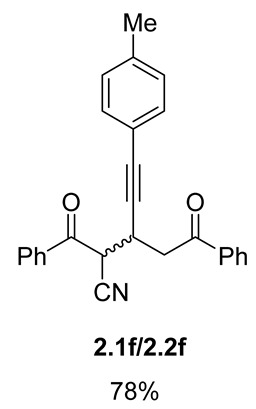
9	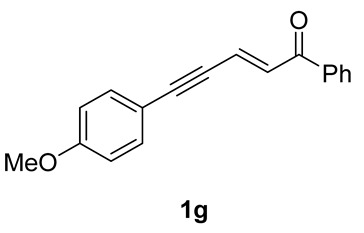	MeONa, MeOH, r.t., 4 h, R = 1:1	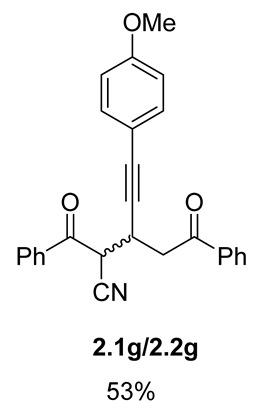
10	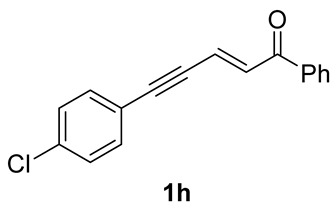	MeONa, MeOH, r.t., 23 h, R = 1:1	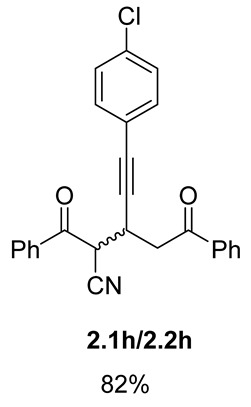
11	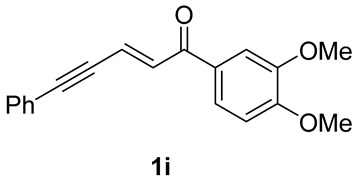	MeONa, MeOH, r.t., 26 h, R=1:1	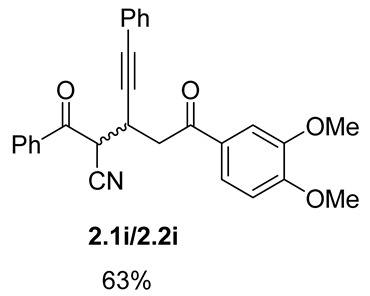
12	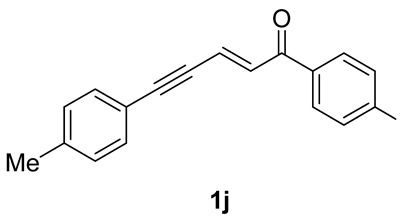	MeONa, MeOH, r.t., 4 h, R= 1:1	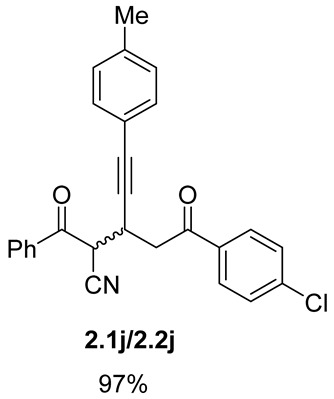
13	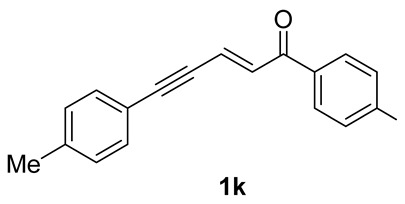	MeONa, MeOH, r.t., 4 h, R = 1:1	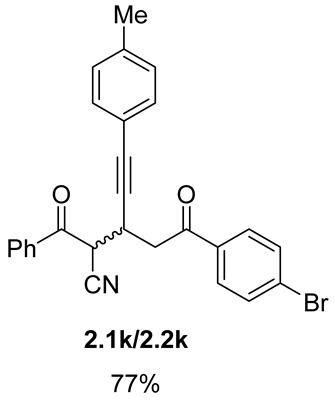
14	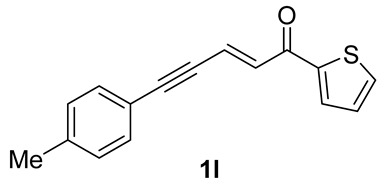	MeONa, MeOH, r.t., 23 h, R = 1:1	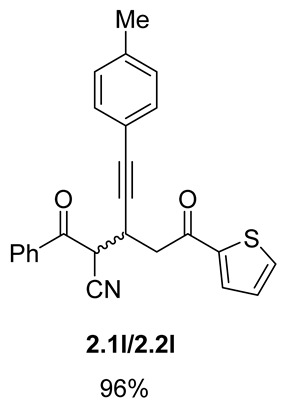

## Data Availability

All the data of the present study are presented in the manuscript. https://www.mdpi.com/ethics.

## Supplementary Materials

The following are available online, ^1^H-, ^13^C-NMR, IR spectra of the obtained compounds **2.1**/**2.2** and **3**.

## Sample Availability

Samples of the compounds **2** are available from the authors.
