# Supplementary material for: Michael Addition of 3-Oxo-3-phenylpropanenitrile to Linear Conjugated Enynones: Approach to Polyfunctional δ-Diketones as Precursors for Heterocycle Synthesis
Source: Molecules. 2022 Feb 13;27(4):1256. doi: 10.3390/molecules27041256 (PMC8877045; doi:10.3390/molecules27041256)
Supplement: Supplementary file 1 [file molecules-27-01256-s001.zip › molecules-1590369-SI.pdf]

Michael addition of 3-oxo-3-phenylpropanenitrile to linear conjugated enynones: approach to polyfunctional δ-diketones as precursors for heterocycle synthesis

Anastasiya V. Igushkina[a], Alexander A. Golovanov[b], Aleksander V. Vasilyev[a,c,]*

[a]*Department of Organic Chemistry, Institute of Chemistry, Saint Petersburg State University, Universitetskaya nab., 7/9, Saint Petersburg, 199034, Russia.*

[b]*Department of Chemistry, Chemical Processes and Technologies, Togliatti State University, Belorusskaya ul., 14, Togliatti, 445667, Russia.*

[c]*Department of Chemistry, Saint Petersburg State Forest Technical University, Institutsky per., 5, Saint Petersburg, 194021, Russia*

*Corresponding author: A.V. Vasilyev; e-mails: *aleksvasil@mail.ru; a.vasilyev@spbu.ru*

Contents

1. ^{1}H, ^{13}C, NMR and IR spectra of compounds **2**…..……………...……………………..S2
2. ^{1}H, ^{13}C, HMQC C-H, COSY H-H, NOESY H-H, HMBC C-H, NMR spectra of compound **3**…………………………………………………………………………S20

1. ^{1}H, NOESY H-H, ^{13}C, NMR and IR spectra of compounds **2**.

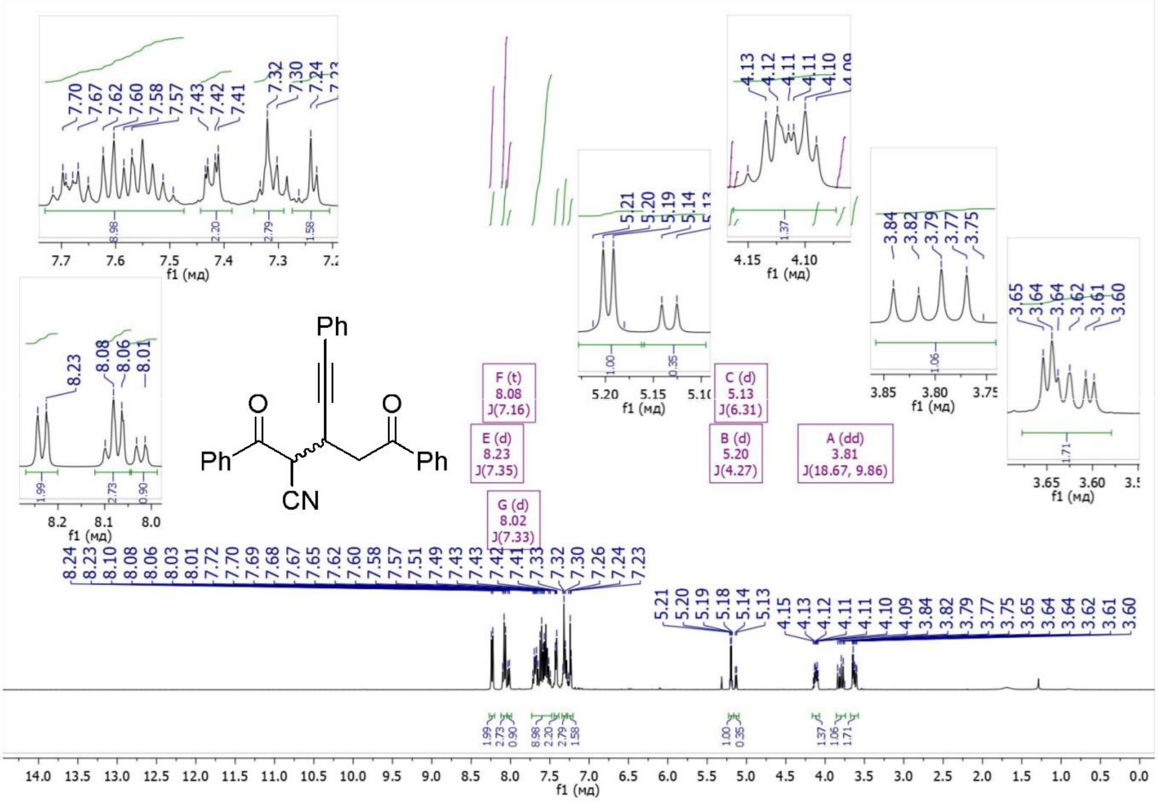

Figure S1. ^{1}H NMR spectrum of the compounds **2.1a/2.2a** ($CDCl_3$, 400 MHz).

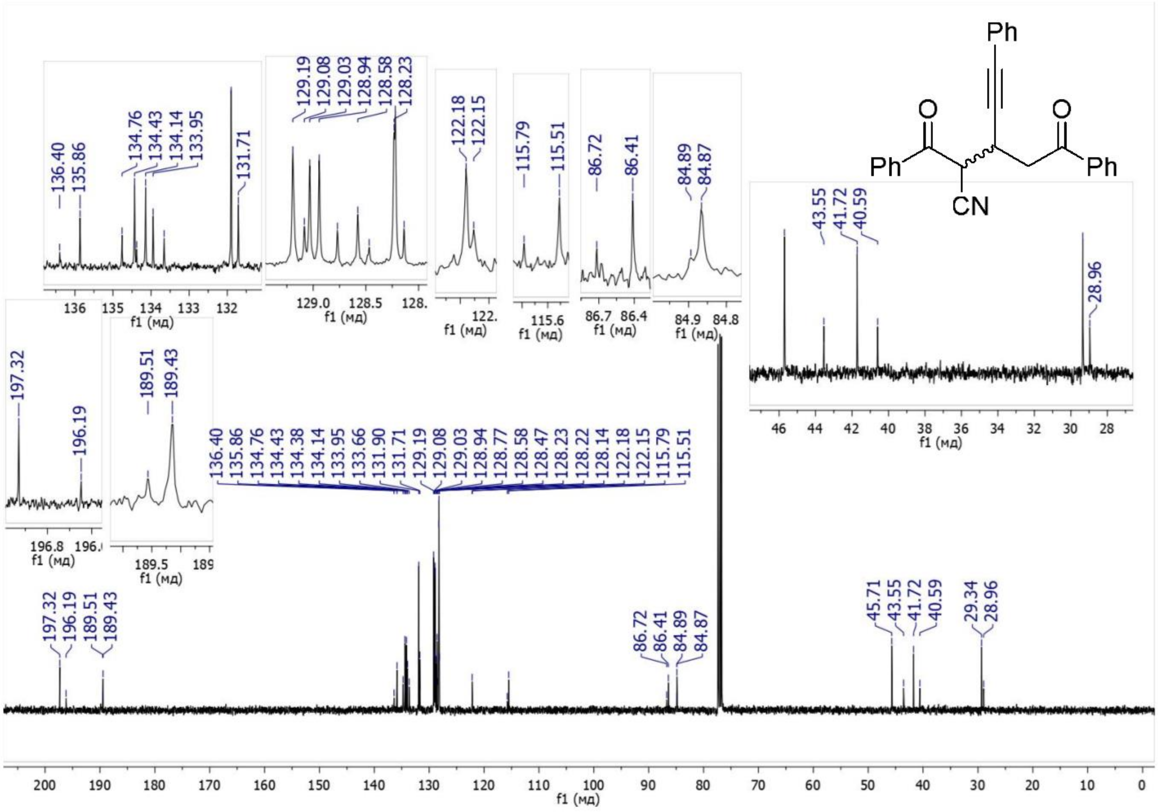

Figure S2. ^{13}C NMR spectrum of the compounds **2.1a/2.2a** ($CDCl_3$, 100 MHz).

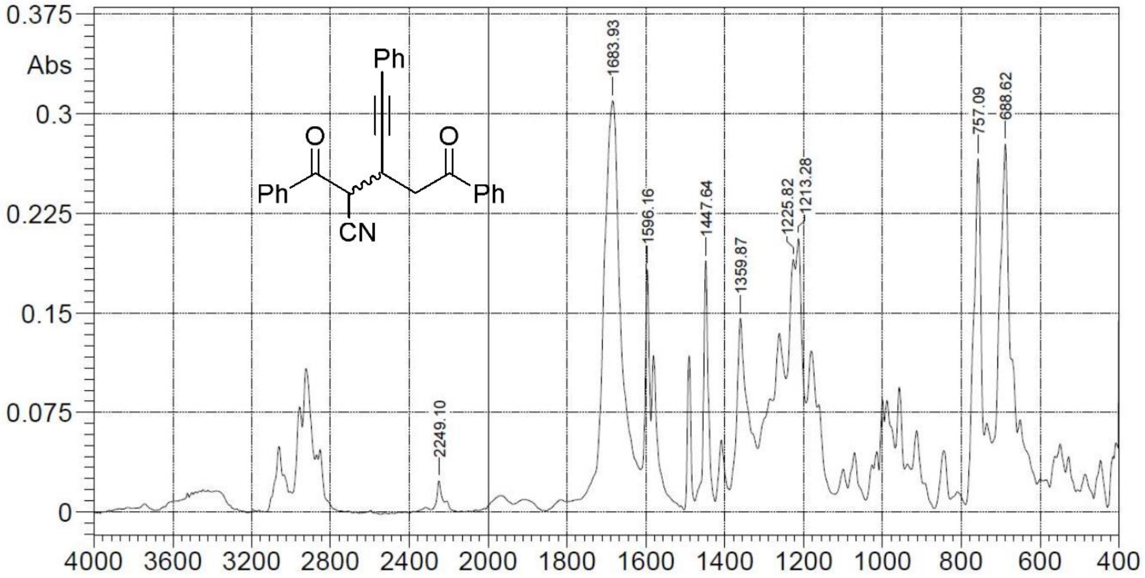

Figure S3. IR of the compound **2.1a/2.2a** (KBr).

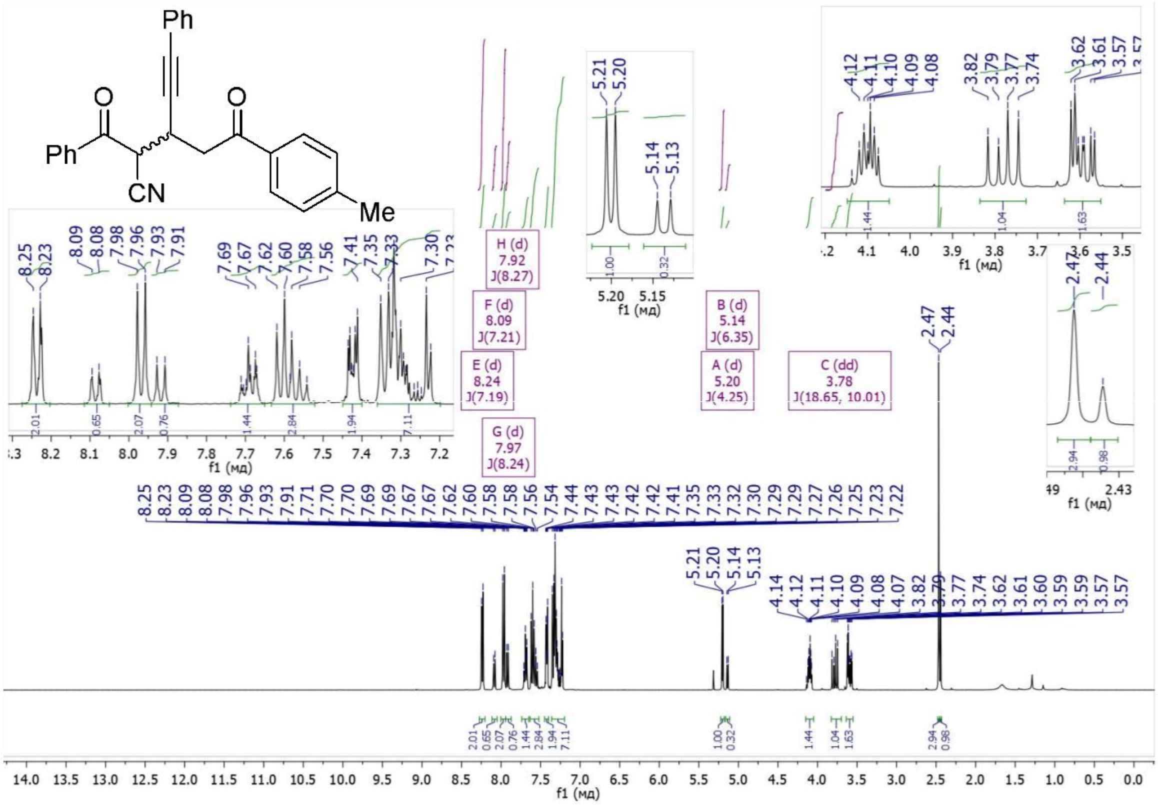

Figure S4. ^{1}H NMR spectrum of the compounds **2.1b/2.2b** ($CDCl_3$, 400 MHz).

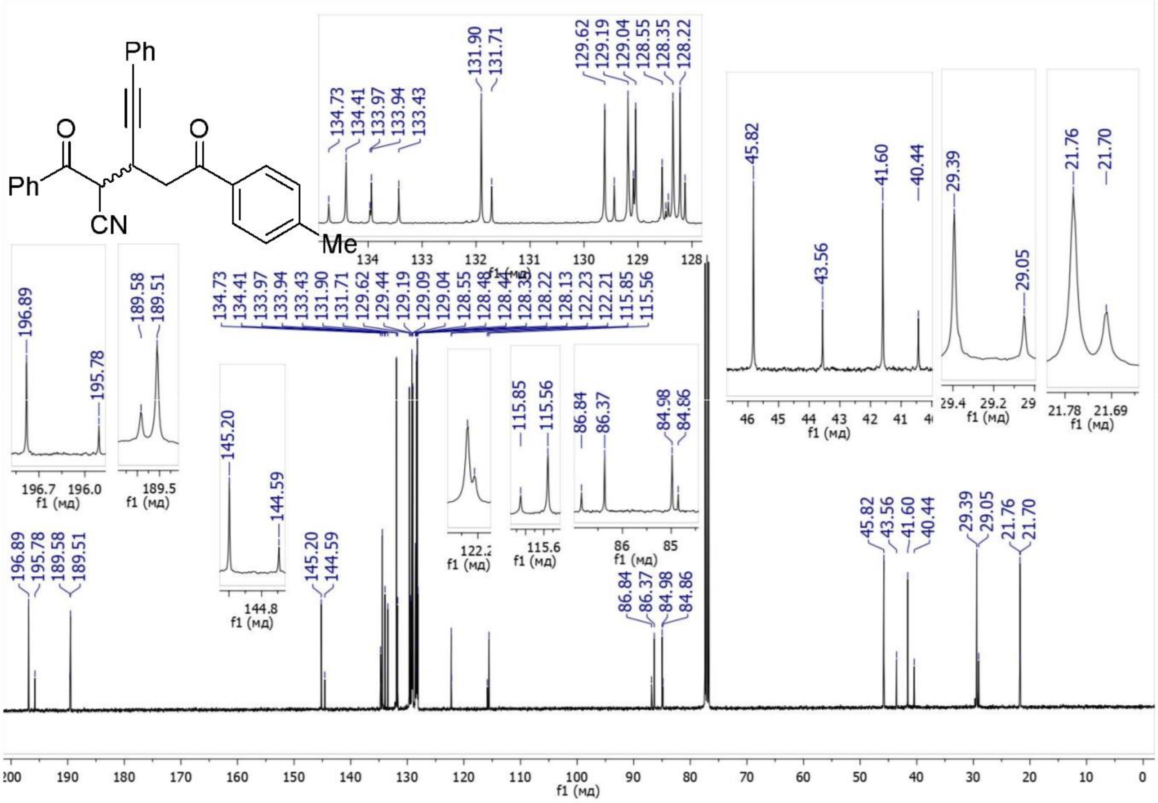

Figure S5. ^{13}C NMR spectrum of the compounds **2.1b/2.2b** ($CDCl_3$, 100 MHz).

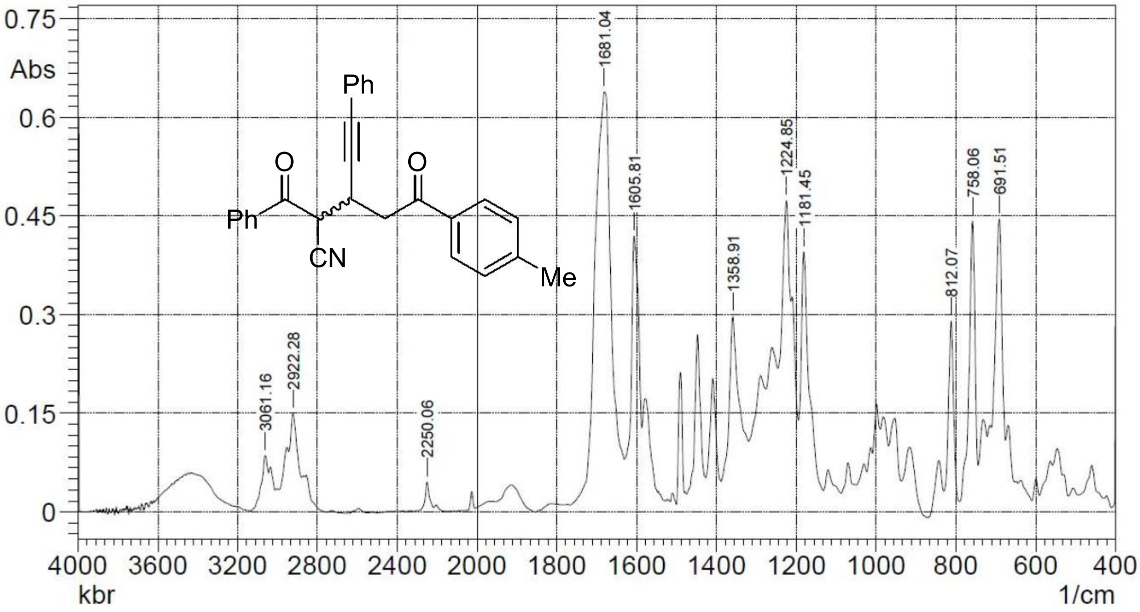

Figure S6. IR of the compound **2.1b/2.2b** (KBr).

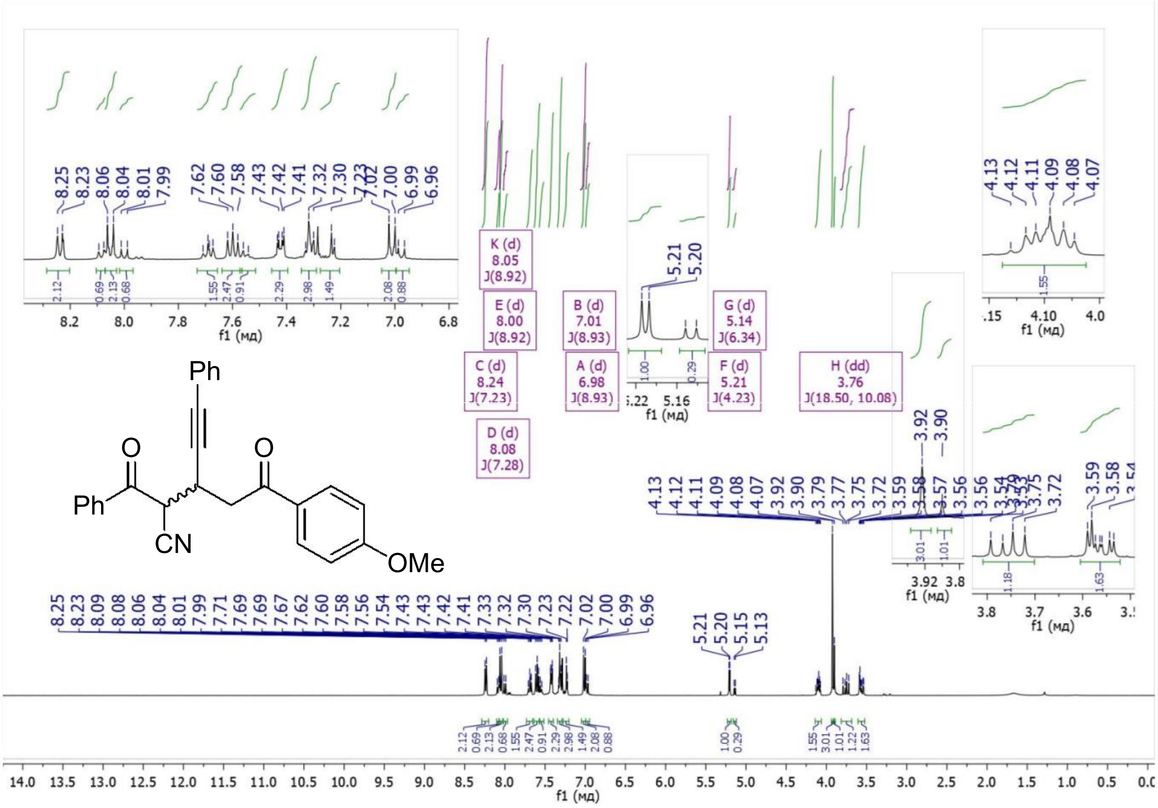

Figure S7. ^{1}H NMR spectrum of the compounds **2.1c/2.2c** ($CDCl_3$, 400 MHz).

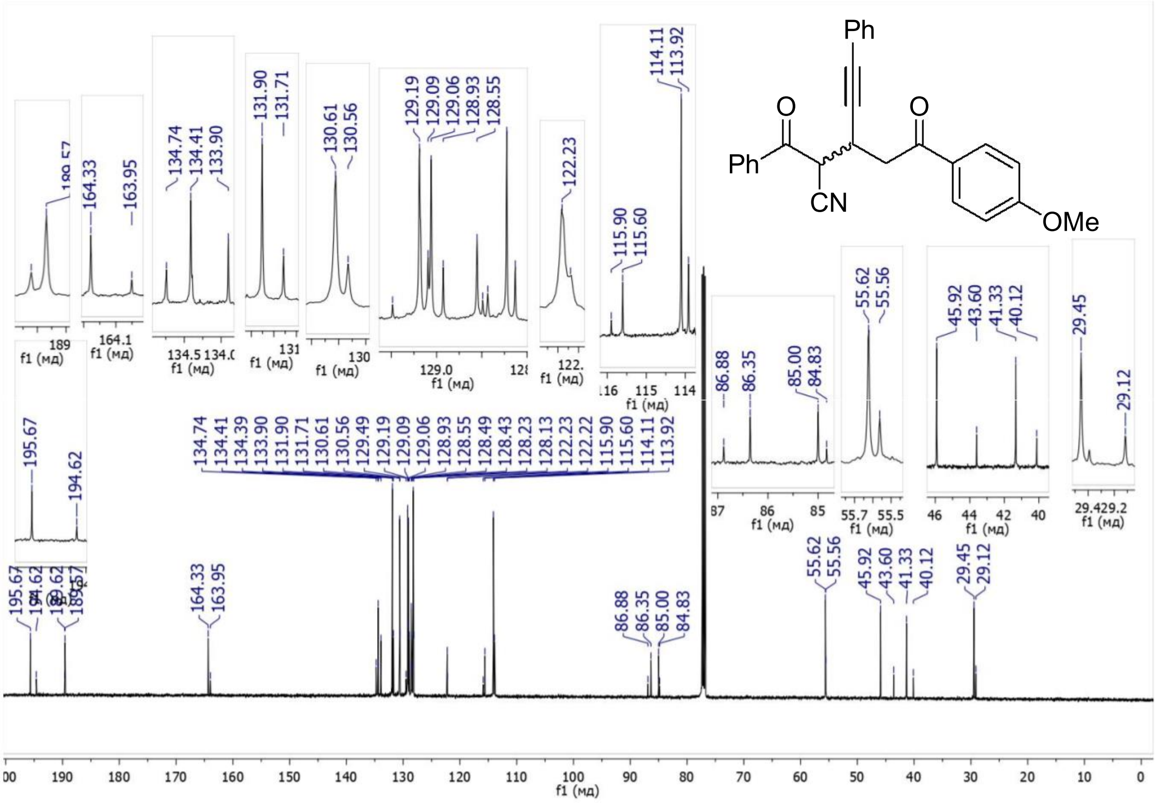

Figure S8. ^{13}C NMR spectrum of the compounds **2.1c/2.2c** ($CDCl_3$, 100 MHz).

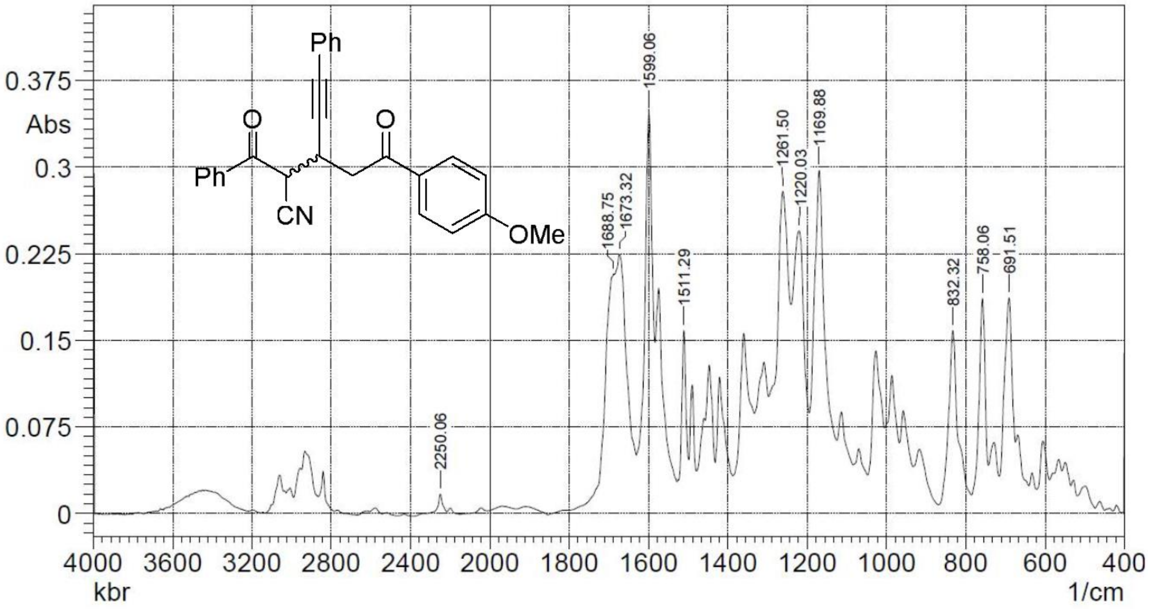

Figure S9. IR of the compound **2.1c/2.2c** (KBr).

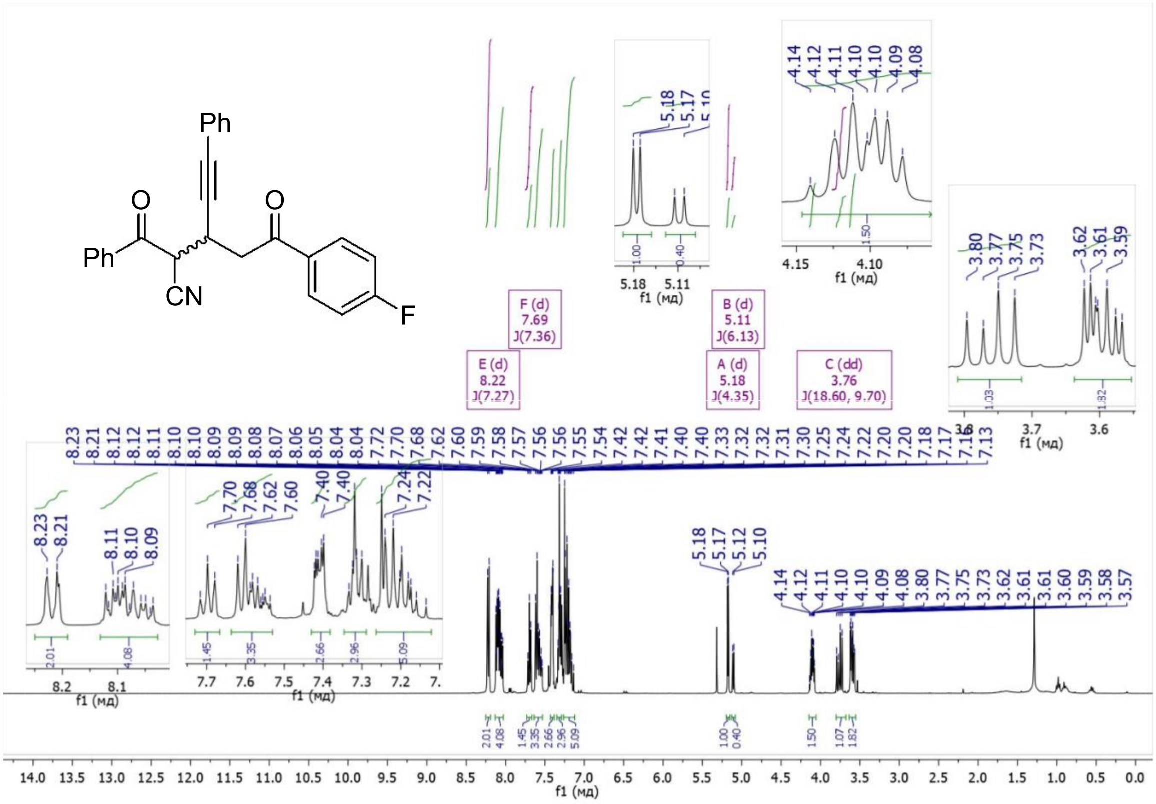

Figure S10. ^{1}H NMR spectrum of the compounds **2.1d/2.2d** ($CDCl_3$, 400 MHz).

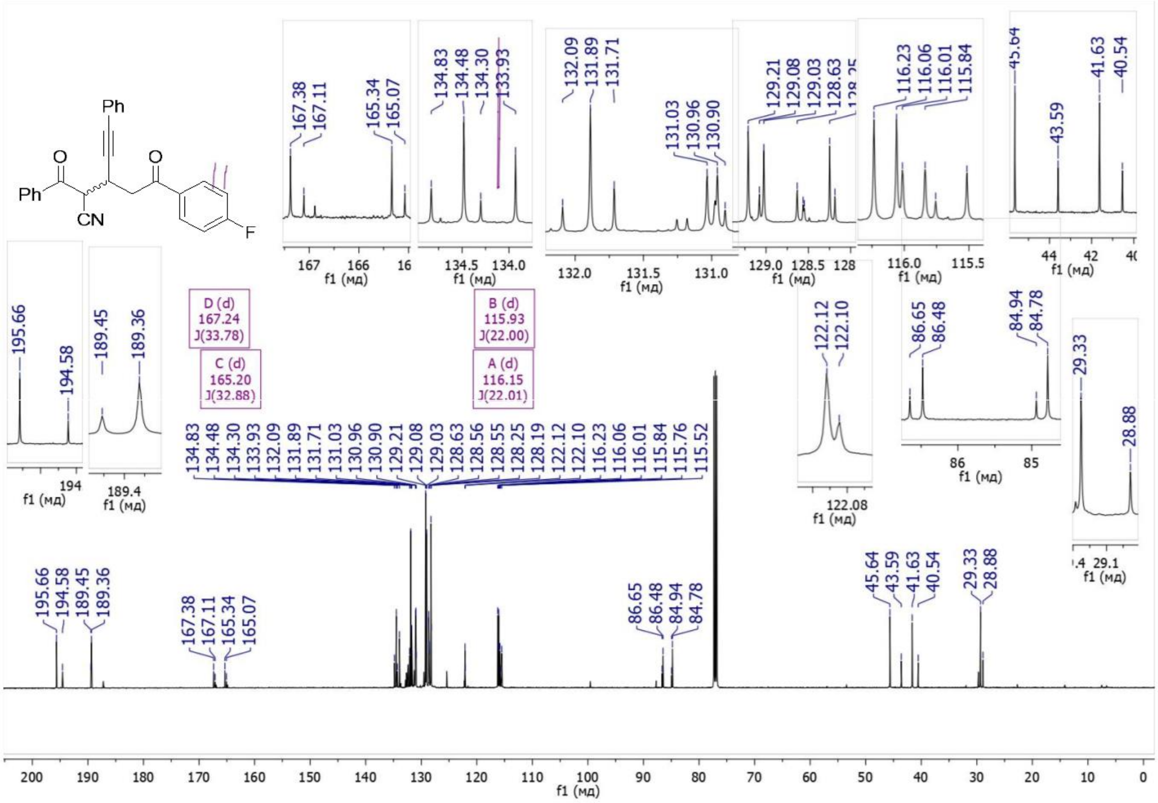

Figure S11. ^{13}C NMR spectrum of the compounds **2.1d/2.2d** ($CDCl_3$, 100 MHz).

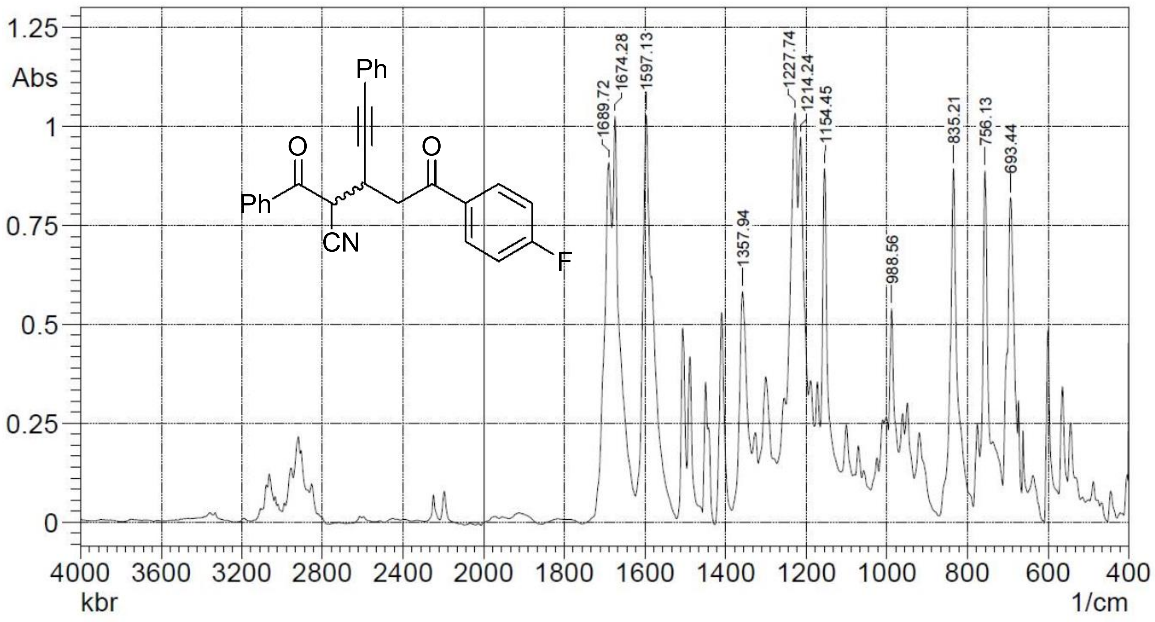

Figure S12. IR of the compound **2.1d/2.2d** (KBr).

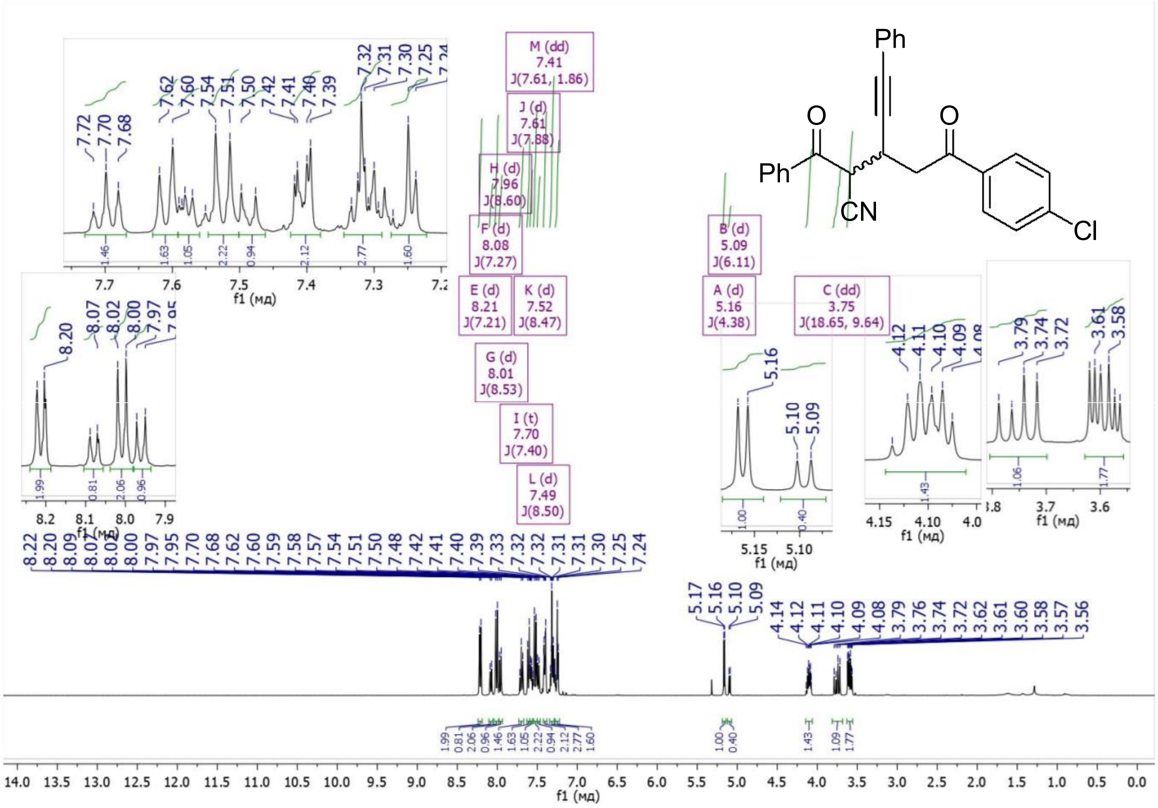

Figure S13. ^{1}H NMR spectrum of the compounds **2.1e/2.2e** ($CDCl_3$, 400 MHz).

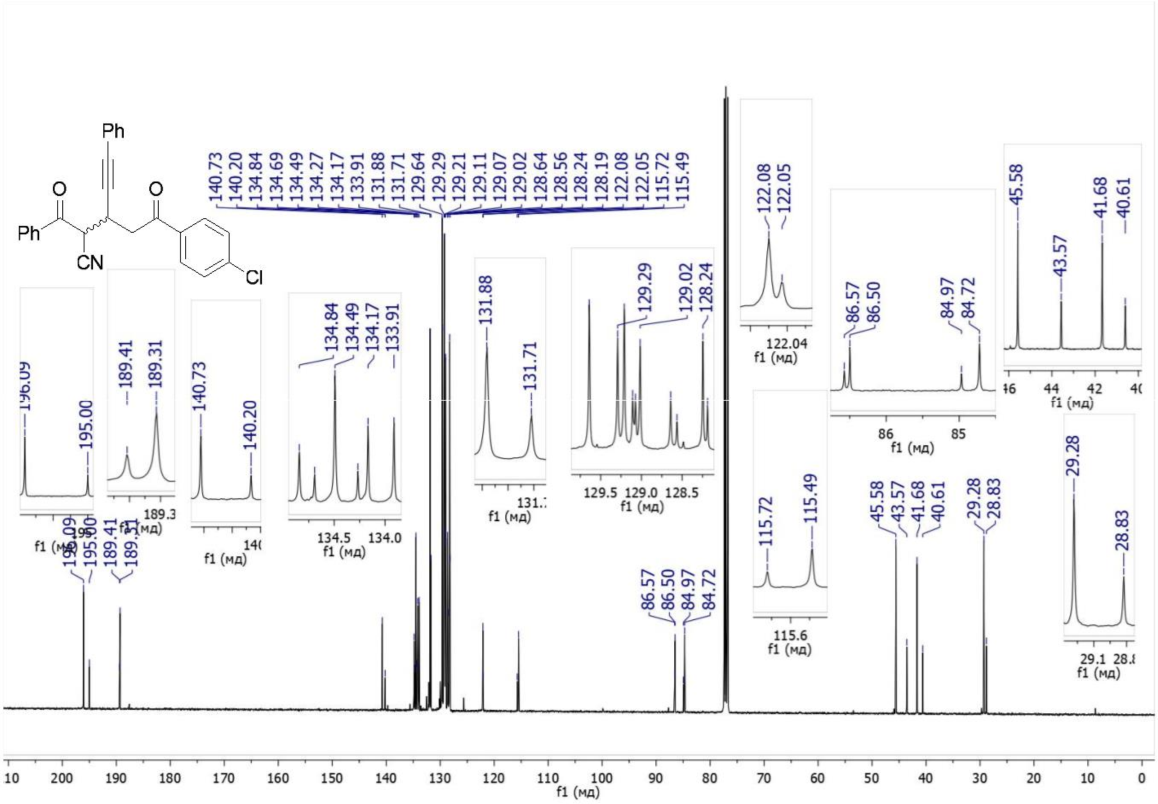

Figure S14. ^{13}C NMR spectrum of the compounds **2.1e/2.2e** ($CDCl_3$, 100 MHz).

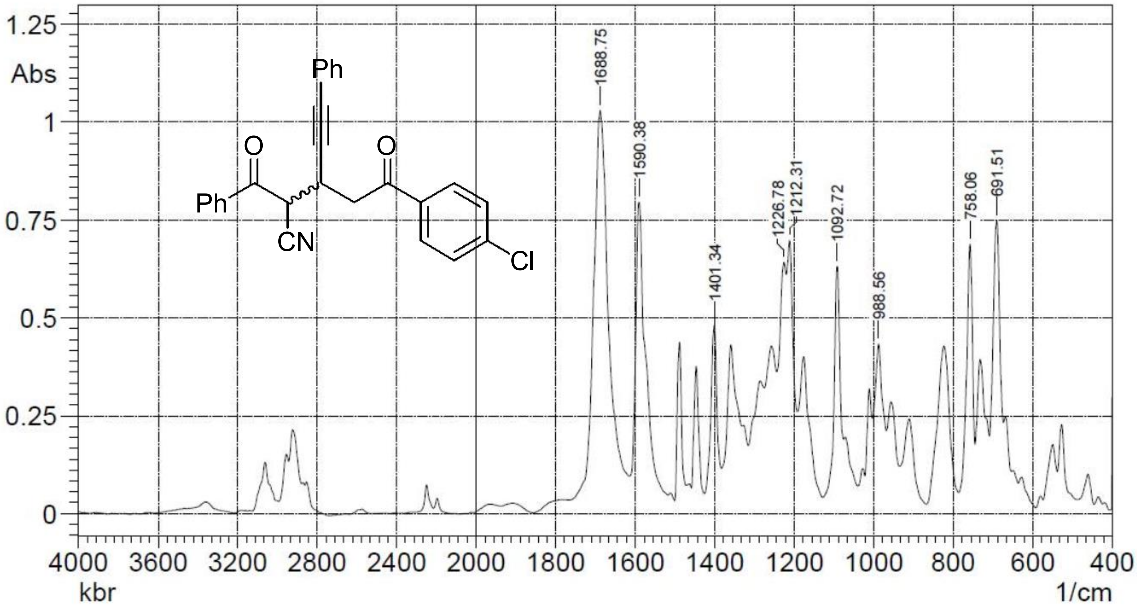

Figure S15. IR of the compound **2.1e/2.2e** (KBr).

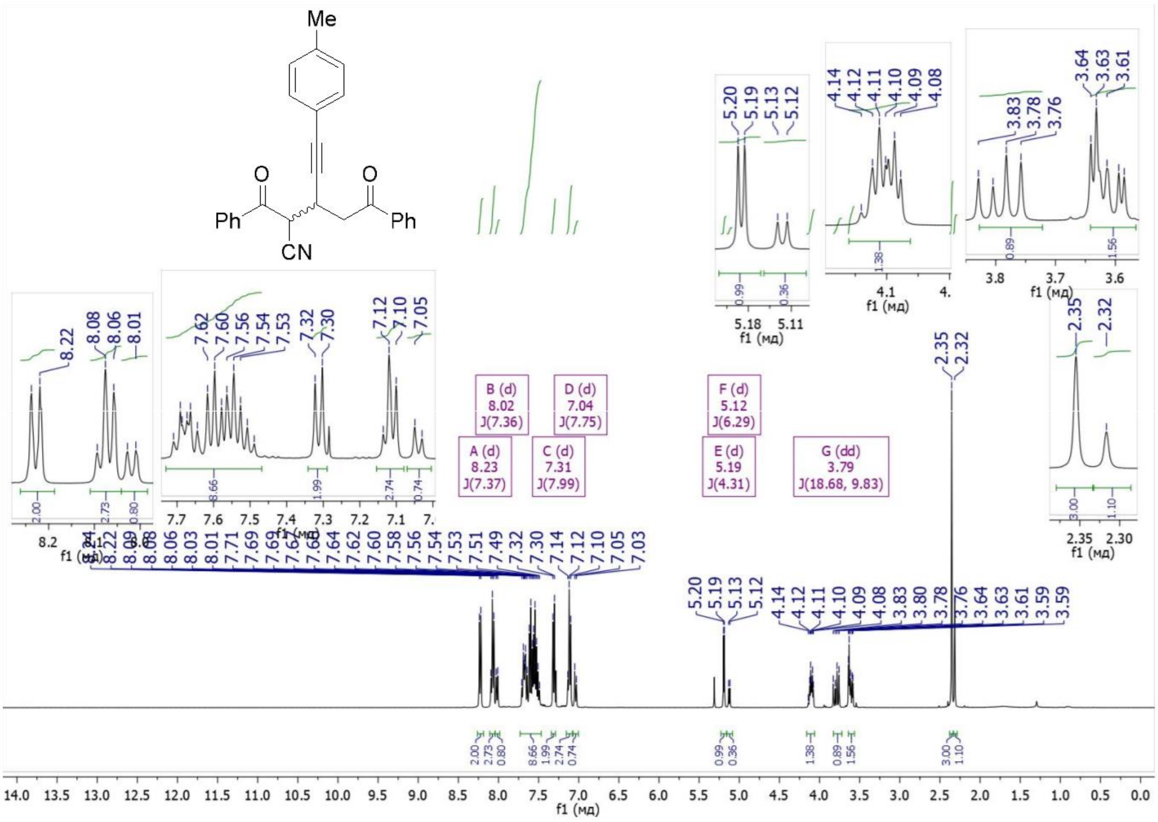

Figure S16. ^{1}H NMR spectrum of the compounds **2.1f/2.2f** ($CDCl_3$, 400 MHz).

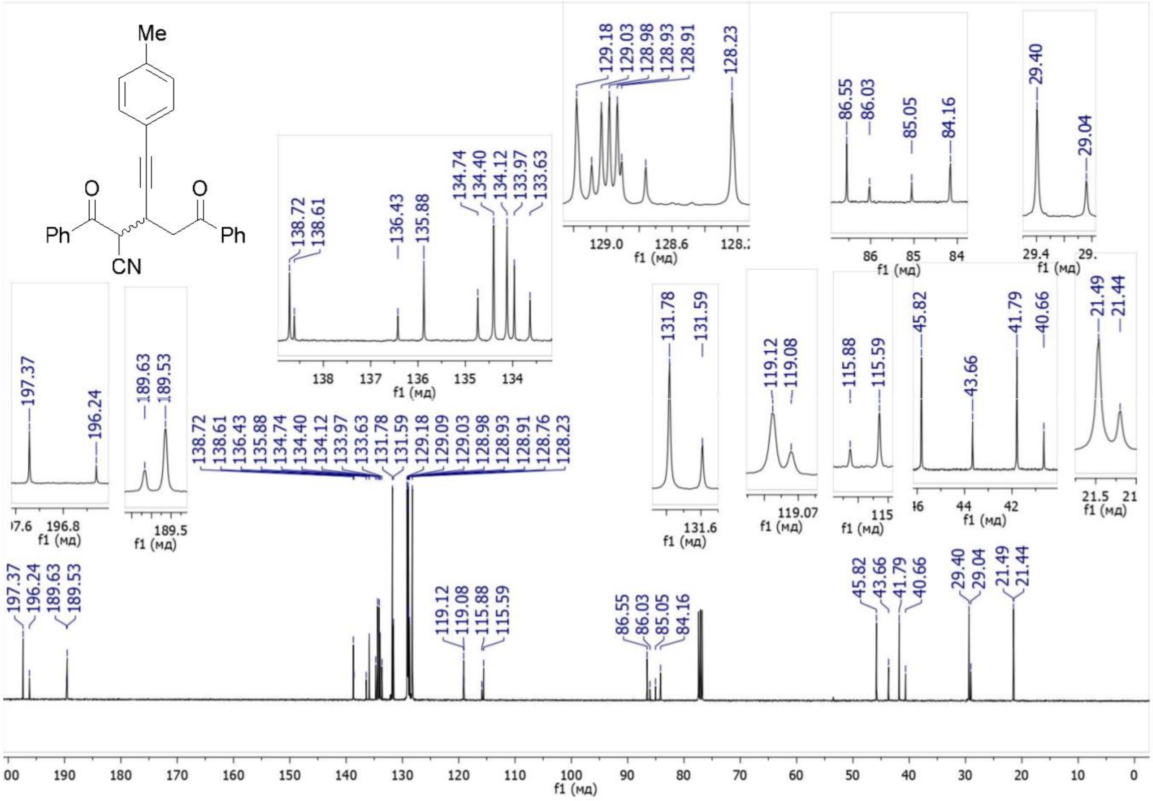

Figure S17. ^{13}C NMR spectrum of the compounds **2.1f/2.2f** ($CDCl_3$, 100 MHz).

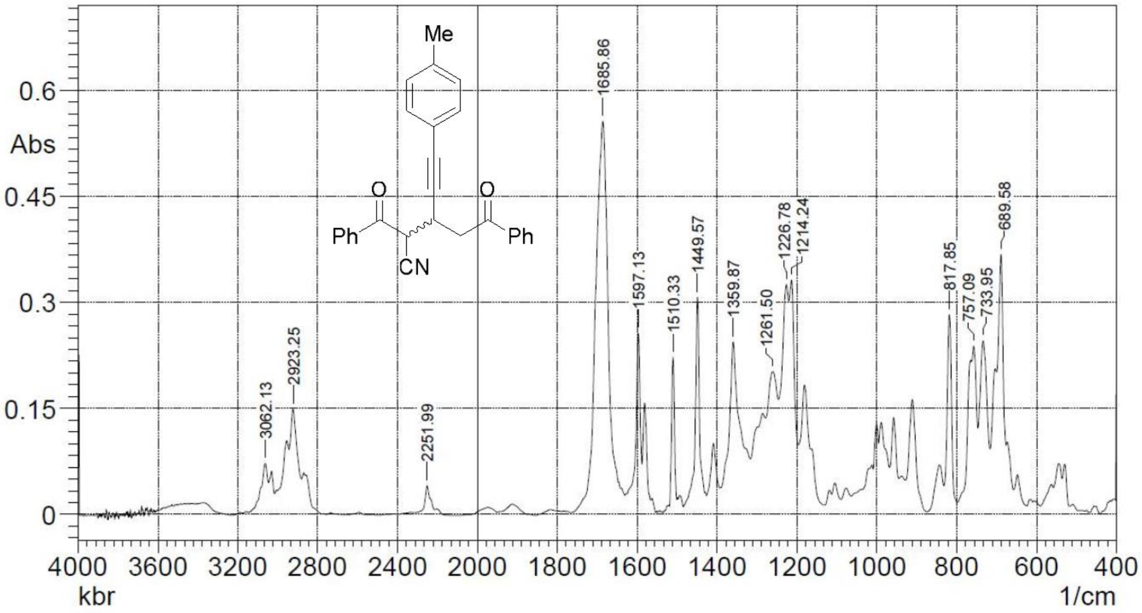

Figure S18. IR of the compound **2.1f/2.2f** (KBr).

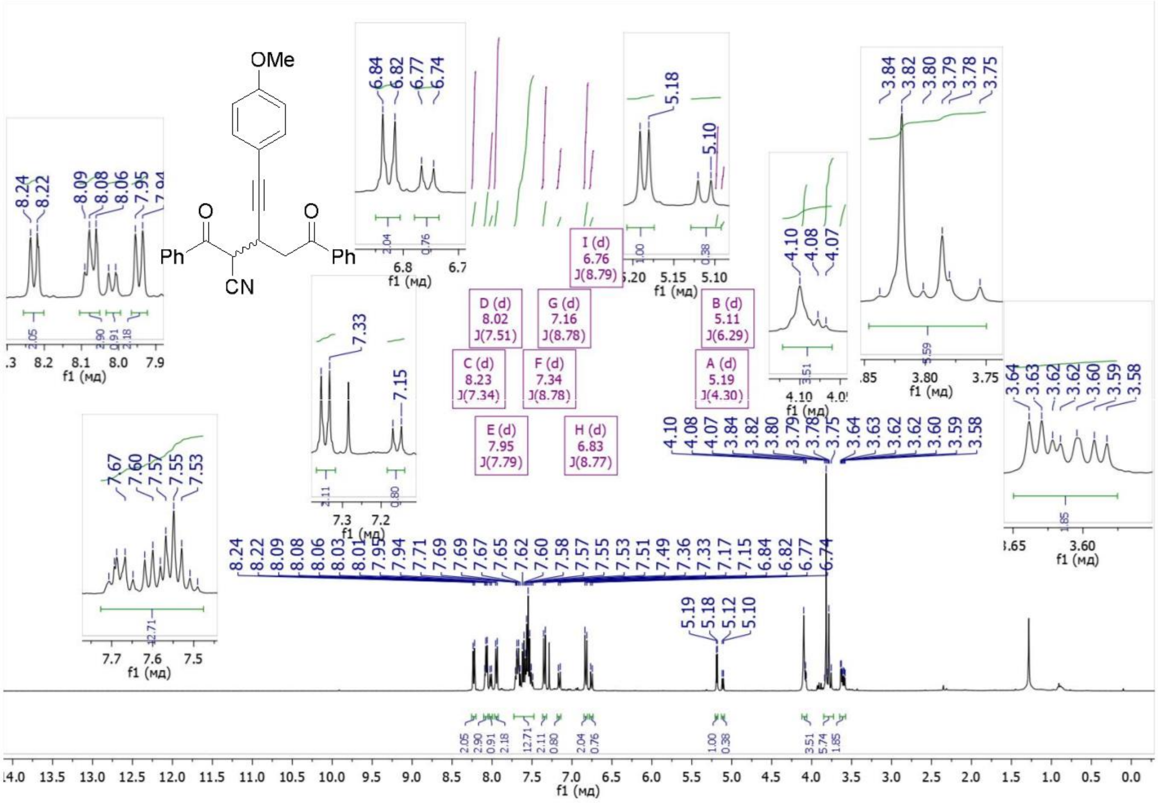

Figure S19. ^{1}H NMR spectrum of the compounds **2.1g/2.2g** ($CDCl_3$, 400 MHz).

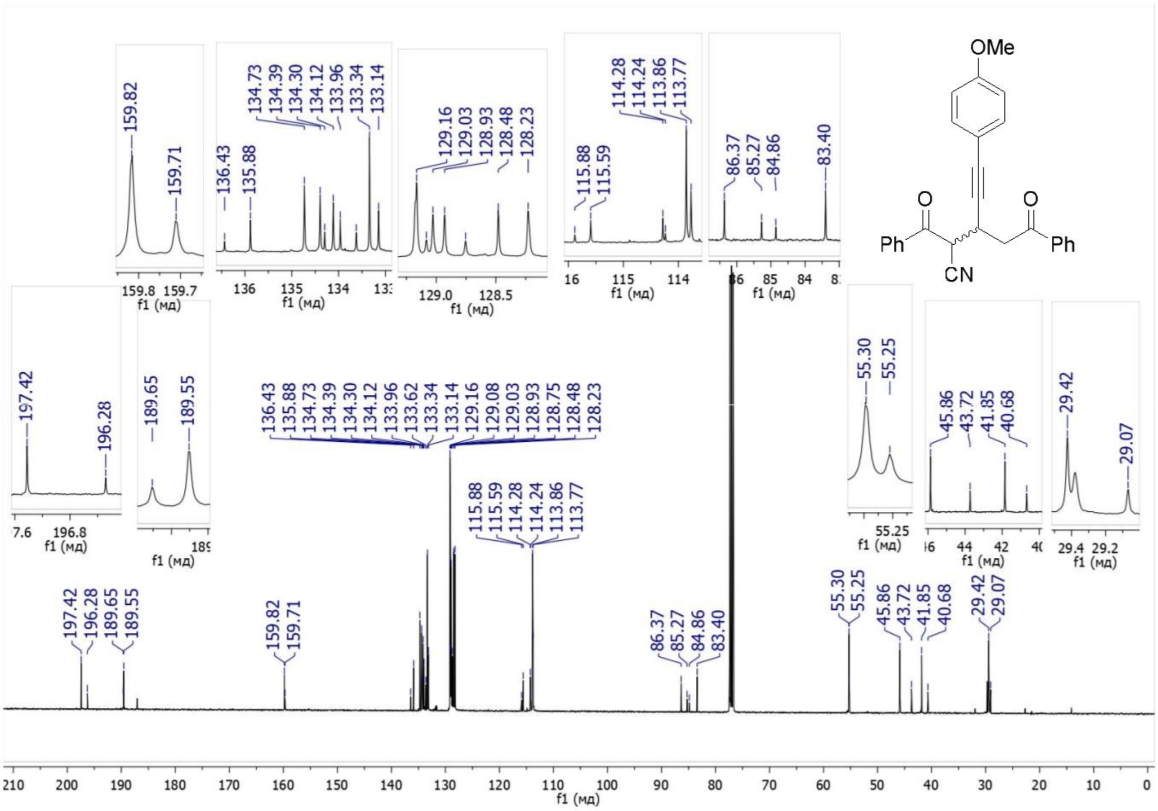

Figure S20. ^{13}C NMR spectrum of the compounds **2.1g/2.2g** ($CDCl_3$, 100 MHz).

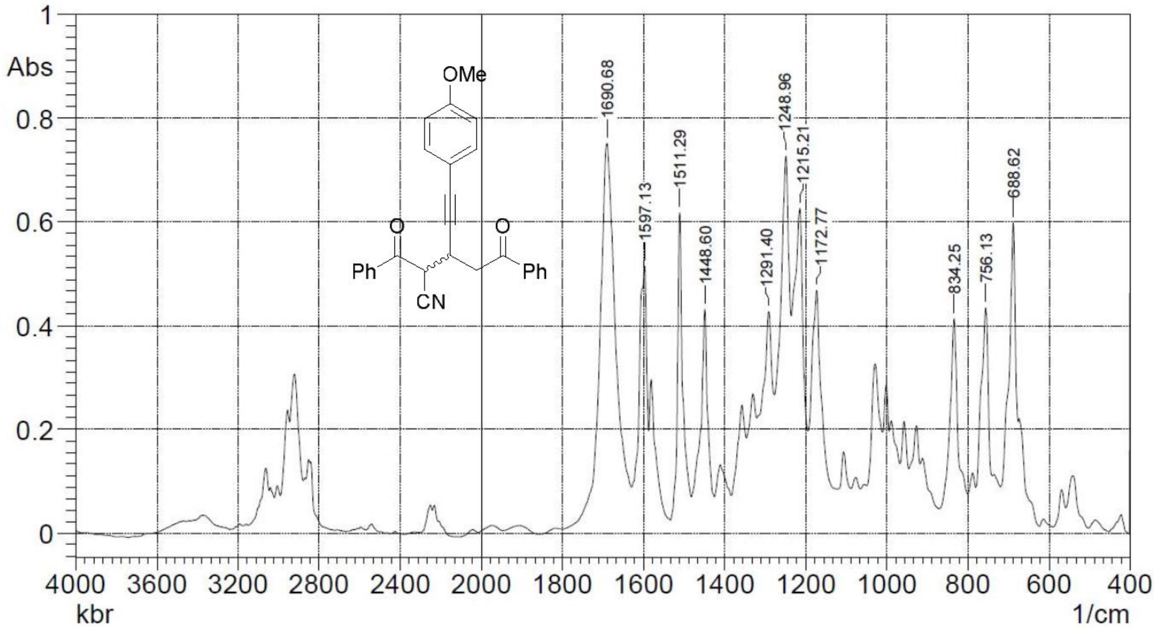

Figure S21. IR of the compound **2.1g/2.2g** (KBr).

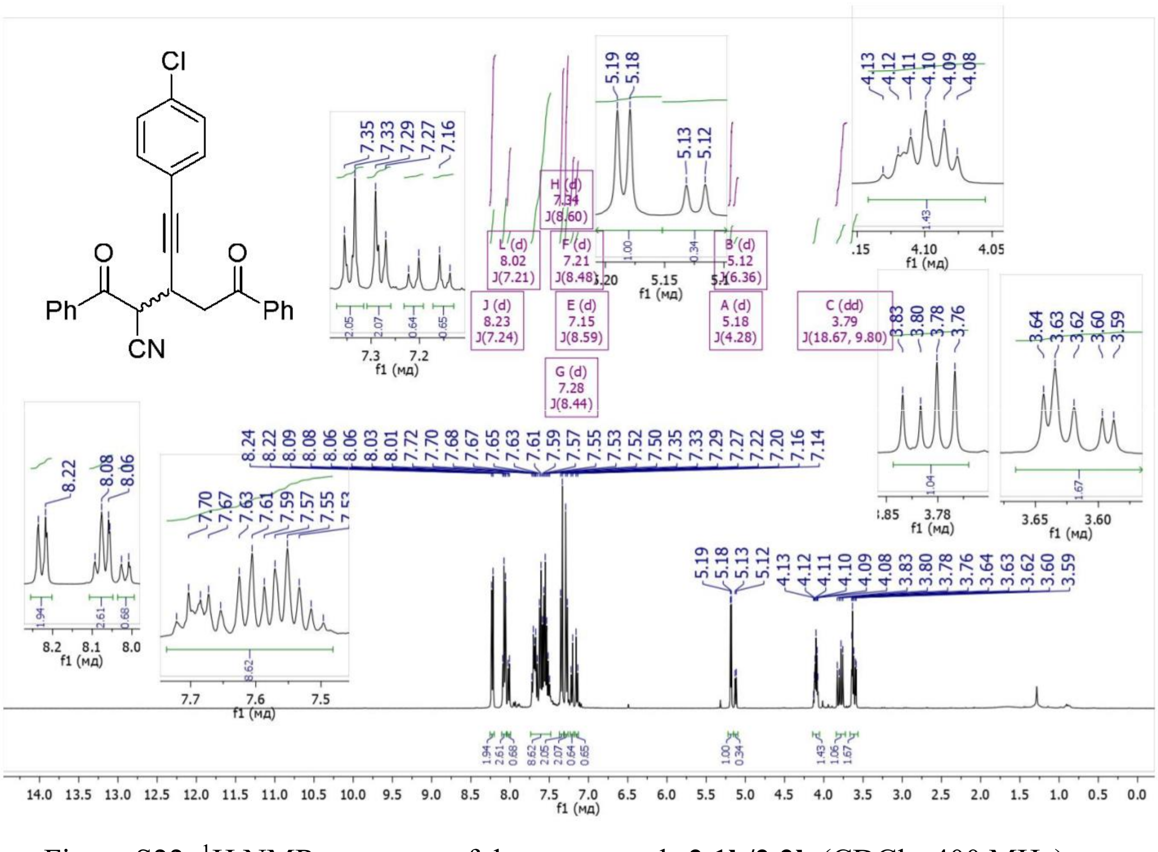

Figure S22. ^{1}H NMR spectrum of the compounds **2.1h/2.2h** ($CDCl_3$, 400 MHz).

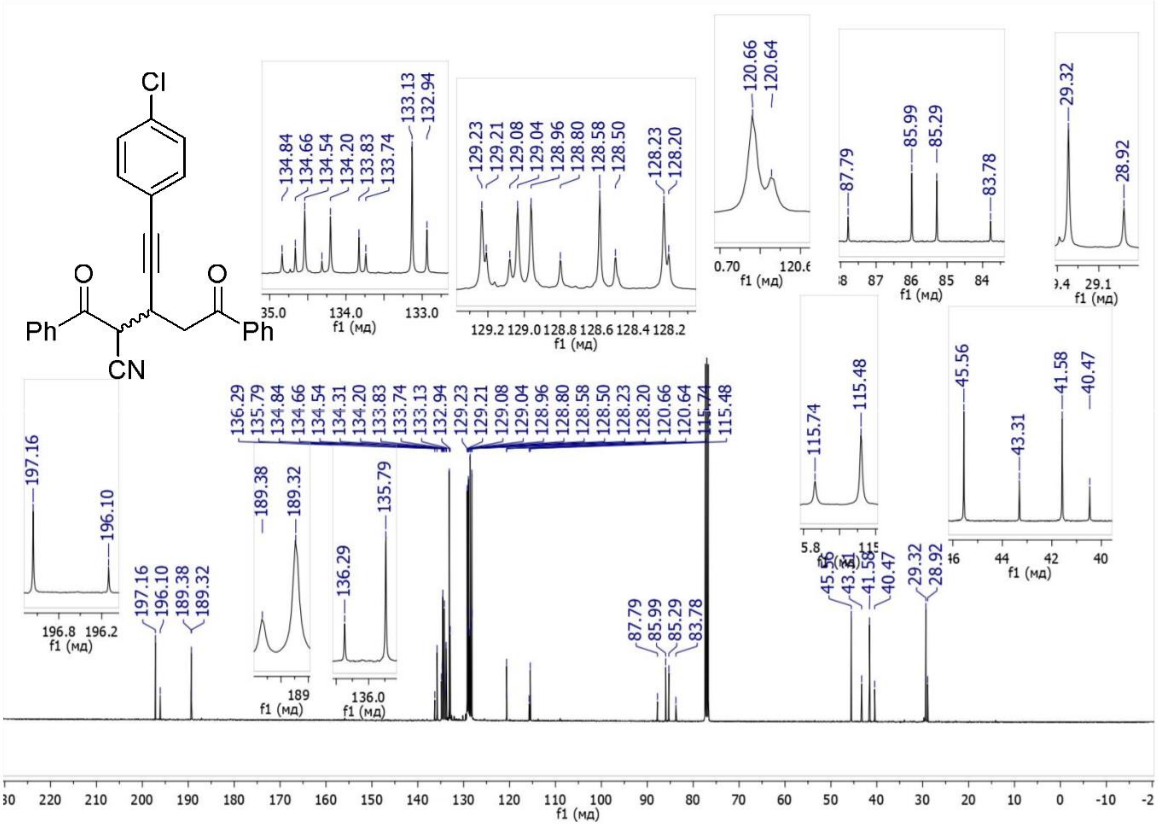

Figure S23. ^{13}C NMR spectrum of the compounds **2.1h/2.2h** ($CDCl_3$, 100 MHz).

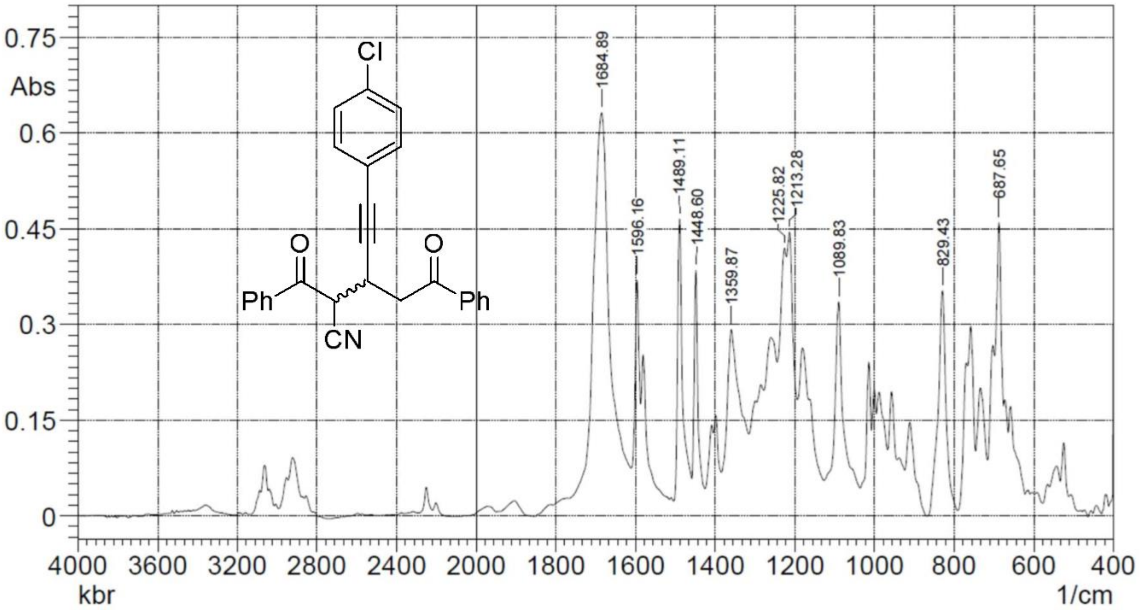

Figure S24. IR of the compound **2.1h/2.2h** (KBr).

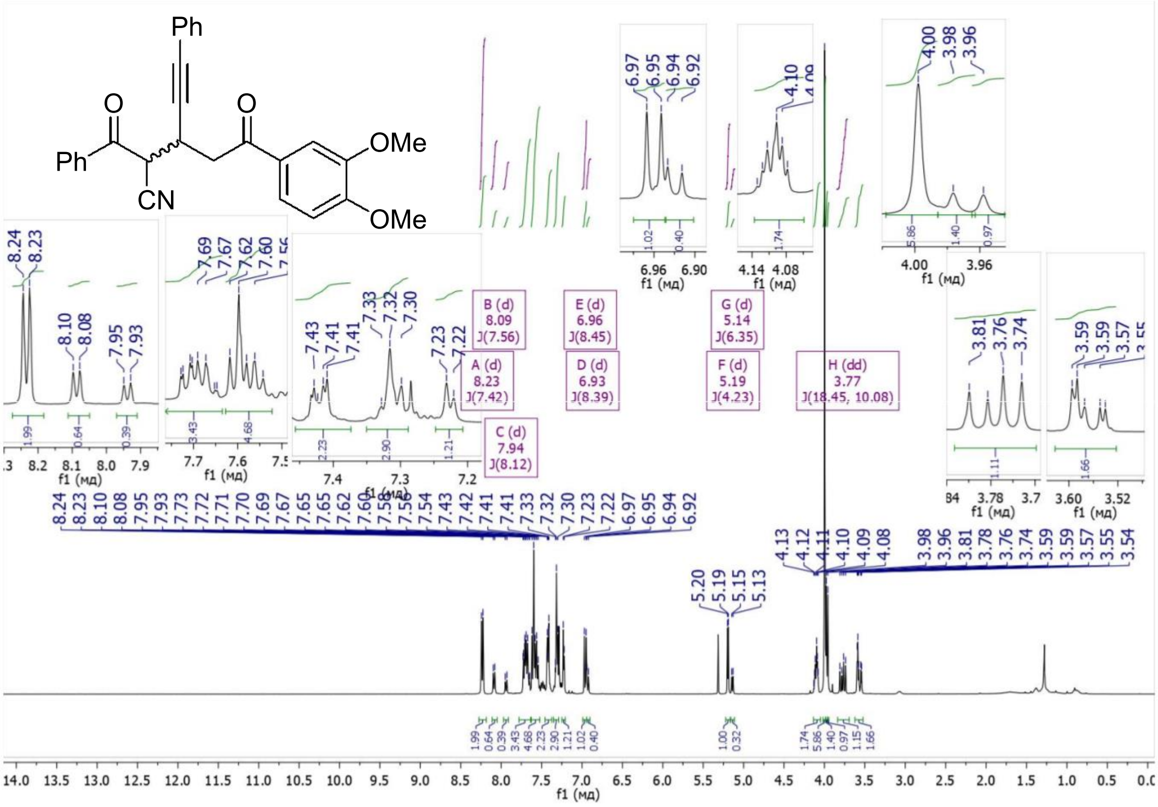

Figure S25. ^{1}H NMR spectrum of the compounds **2.1i/2.2i** ($CDCl_3$, 400 MHz).

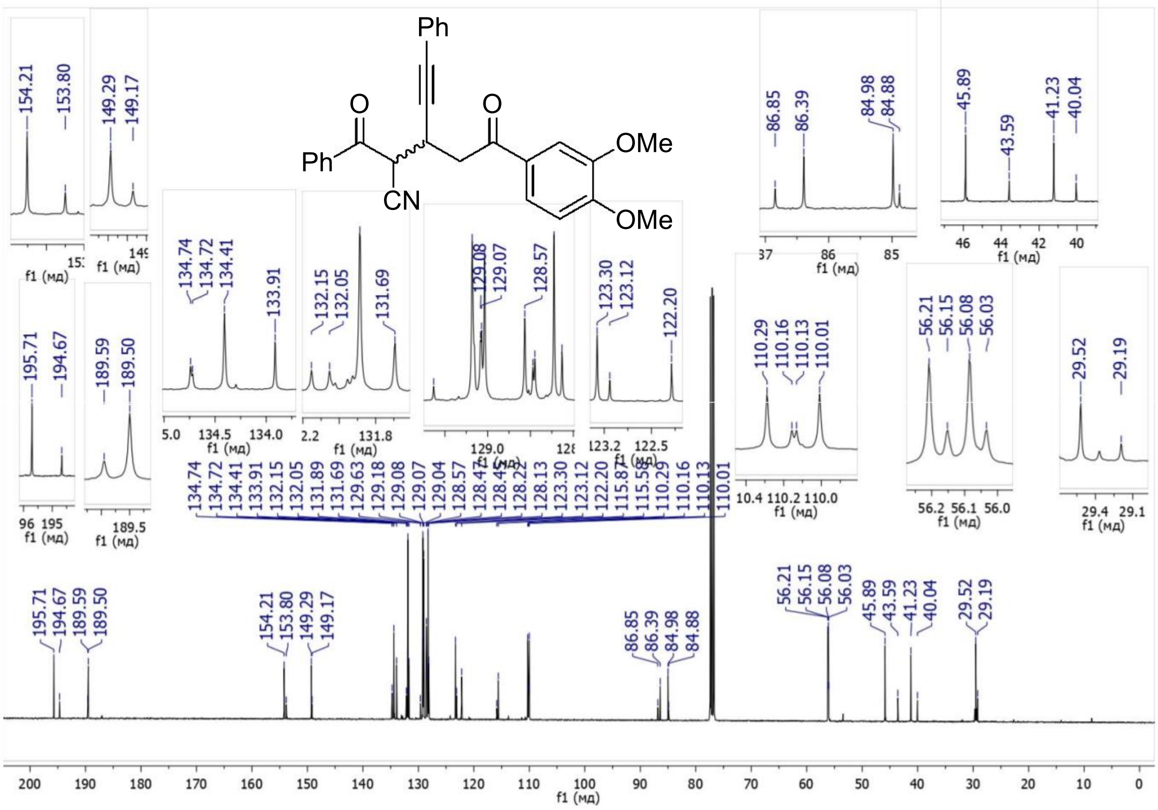

Figure S26. ^{13}C NMR spectrum of the compounds **2.1i/2.2i** ($CDCl_3$, 100 MHz).

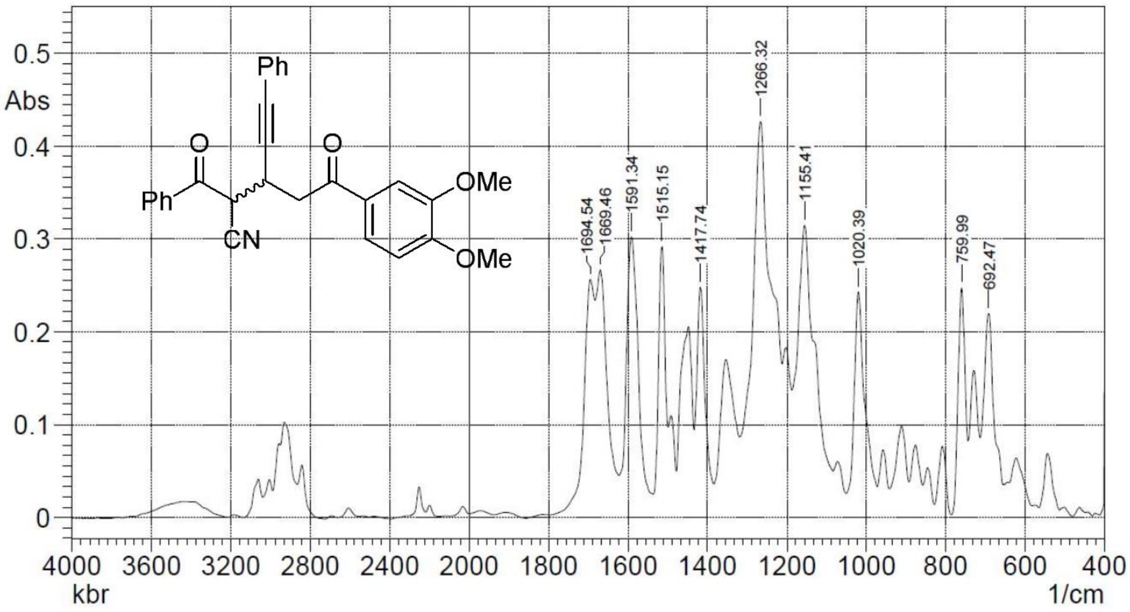

Figure S27. IR of the compound **2.1i/2.2i** (KBr).

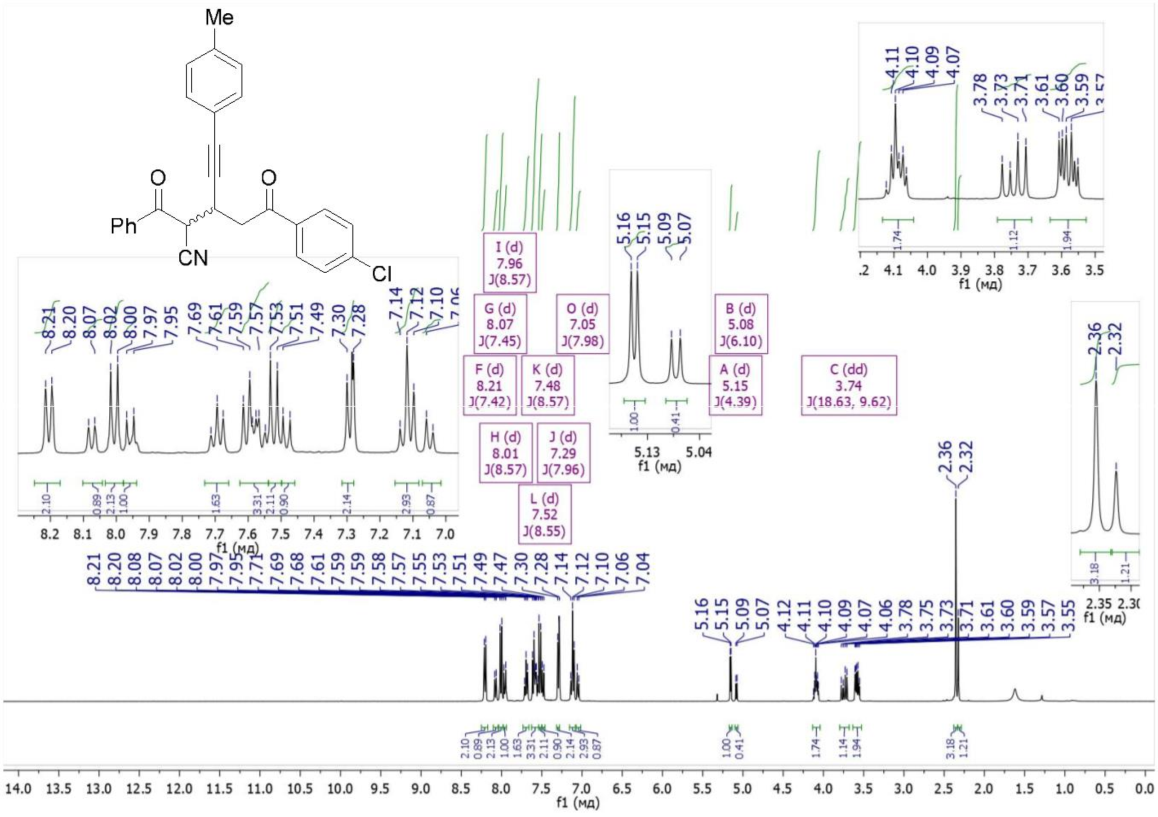

Figure S28. ^{1}H NMR spectrum of the compounds **2.1j/2.2j** ($CDCl_3$, 400 MHz).

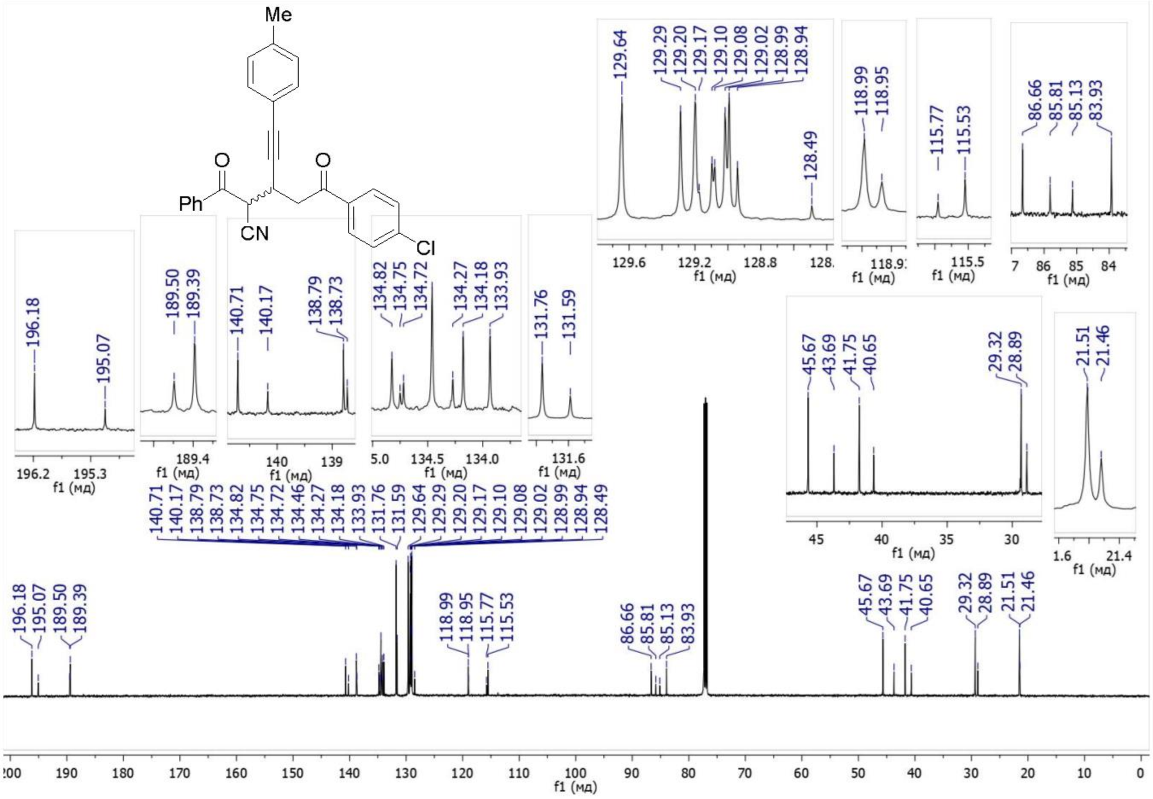

Figure S29. ^{13}C NMR spectrum of the compounds **2.1j/2.2j** ($CDCl_3$, 100 MHz).

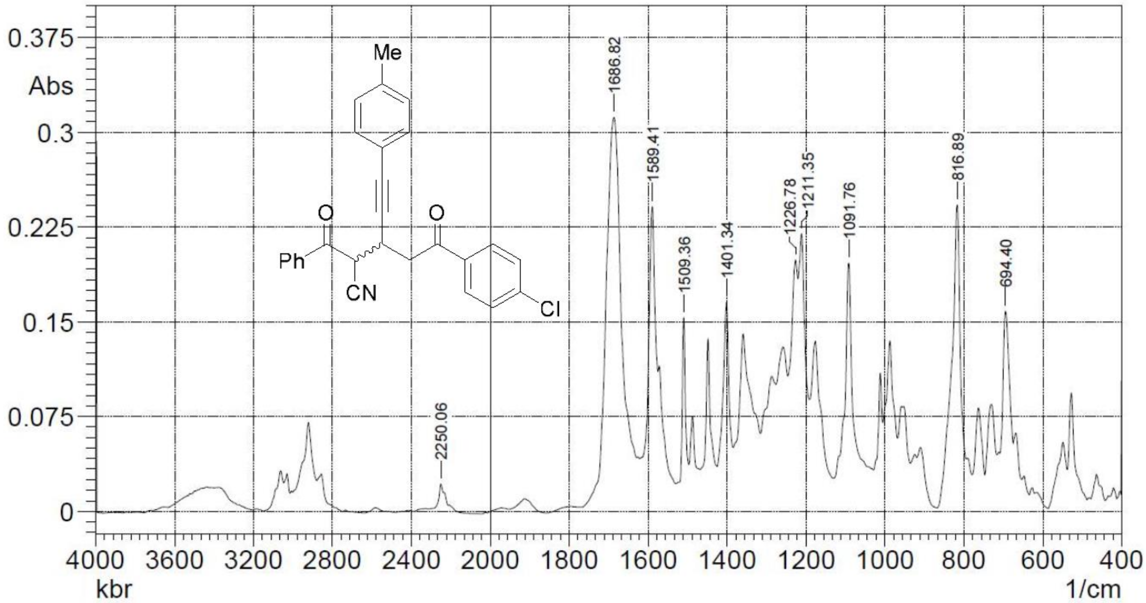

Figure S30. IR of the compound **2.1j/2.2j** (KBr).

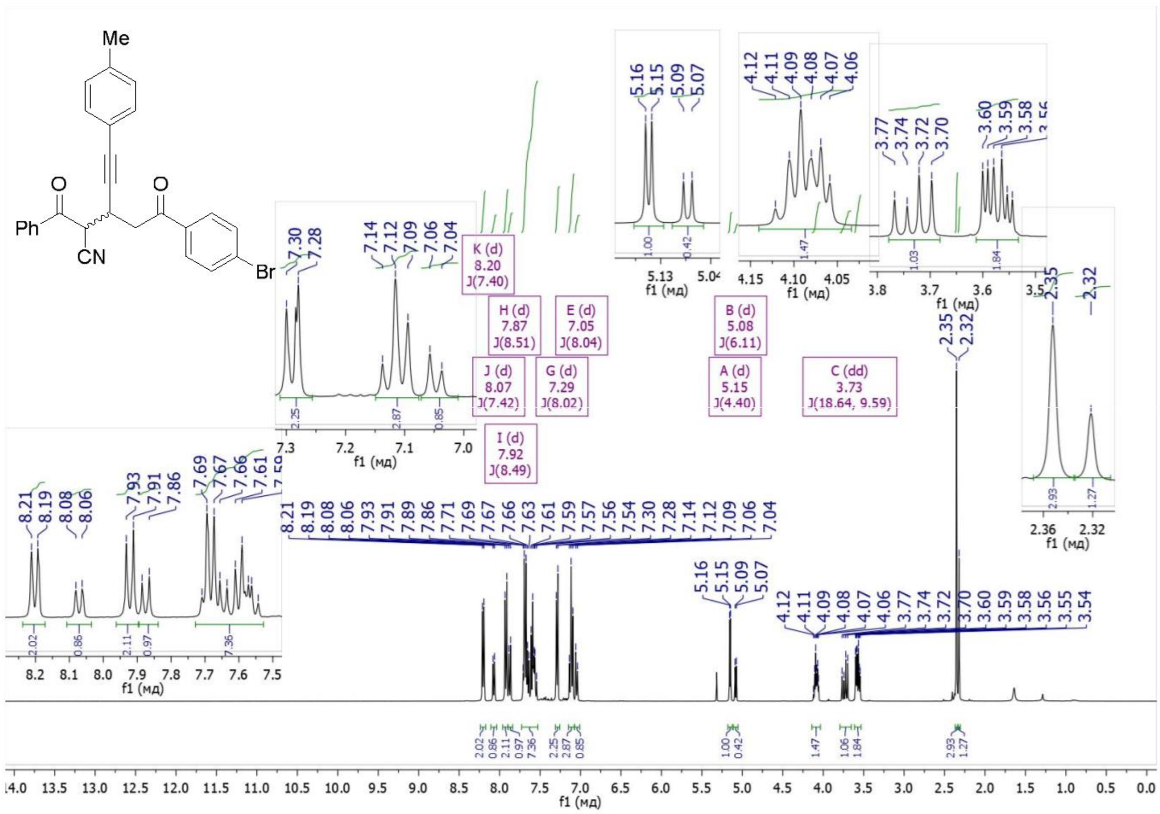

Figure S31. ^{1}H NMR spectrum of the compounds **2.1k/2.2k** ($CDCl_3$, 400 MHz).

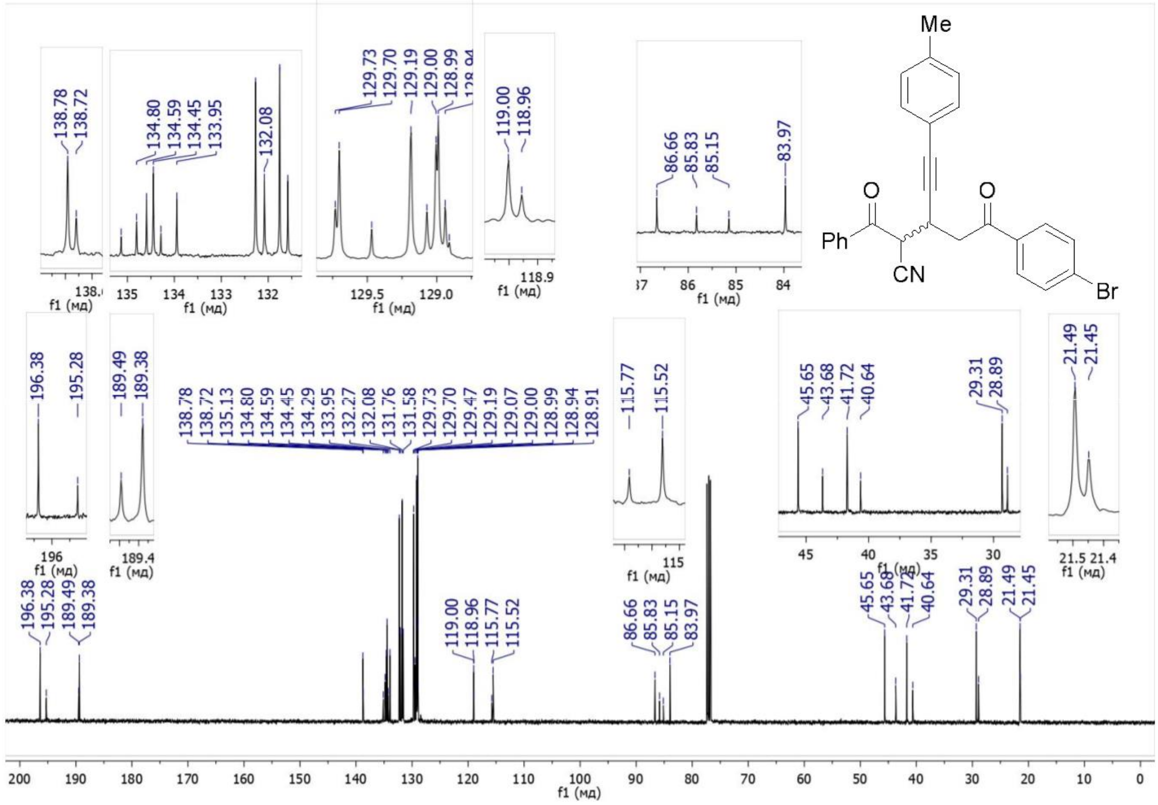

Figure S32. ^{13}C NMR spectrum of the compounds **2.1k/2.2k** ($CDCl_3$, 100 MHz).

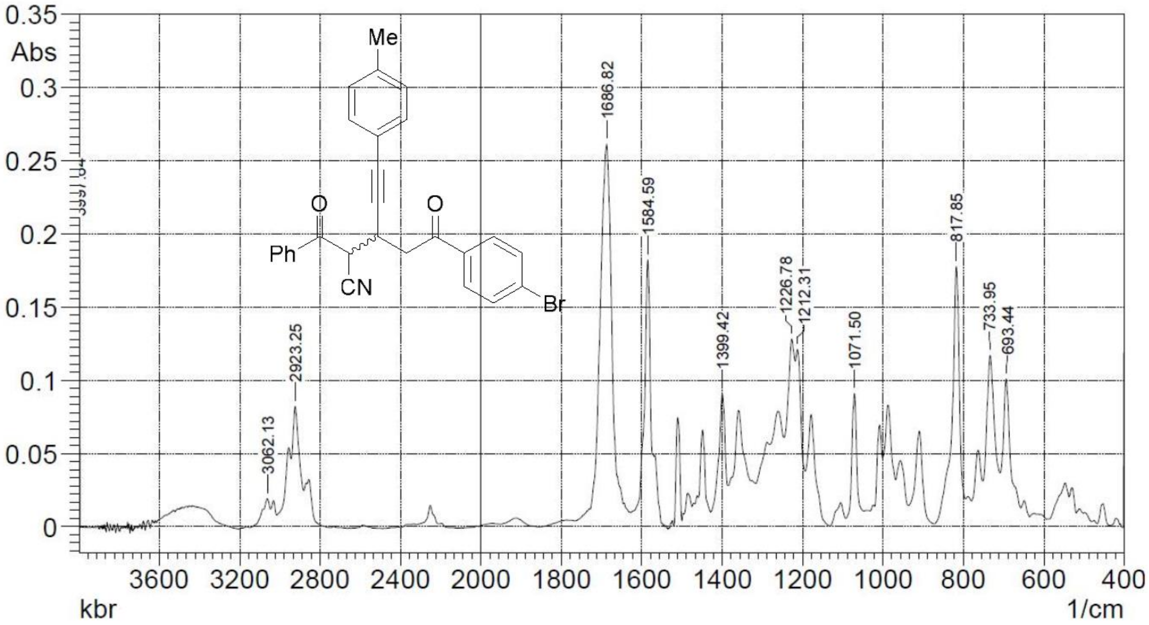

Figure S33. IR of the compound **2.1k/2.2k** (KBr).

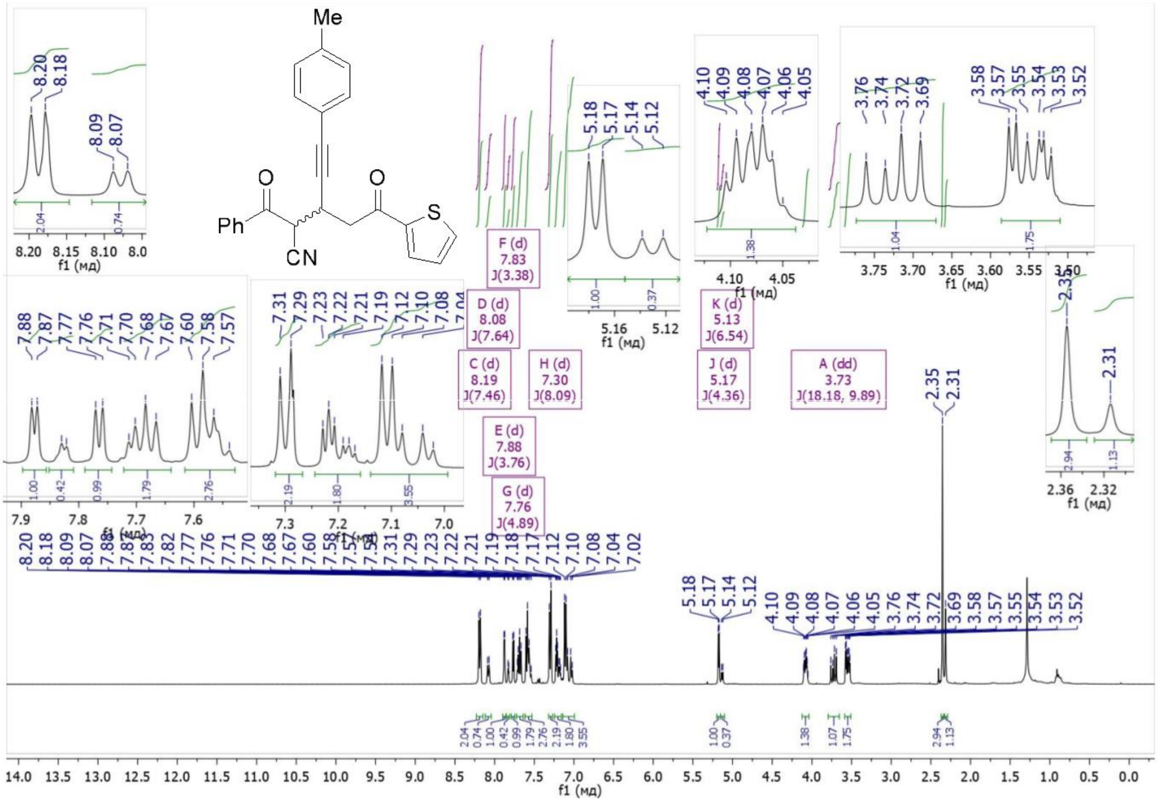

Figure S34. ^{1}H NMR spectrum of the compounds **2.1l/2.2l** ($CDCl_3$, 400 MHz).

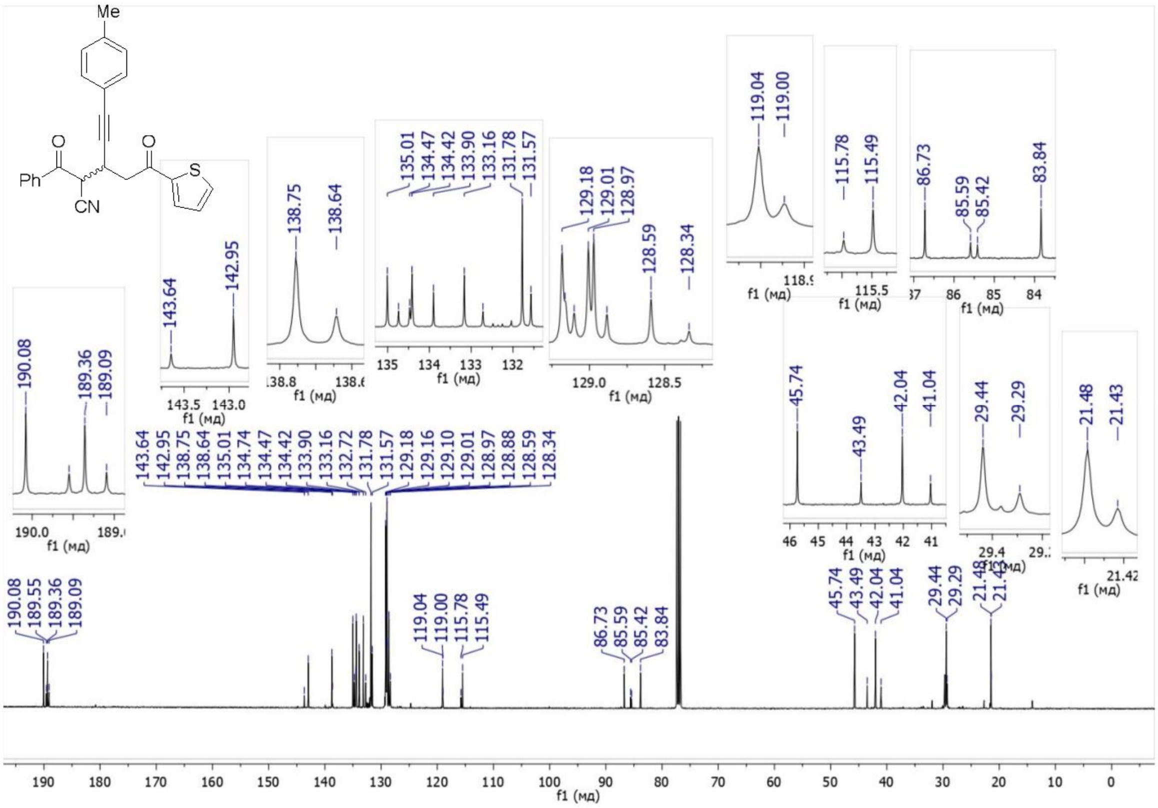

Figure S35. ^{13}C NMR spectrum of the compounds **2.1l/2.2l** ($CDCl_3$, 100 MHz).

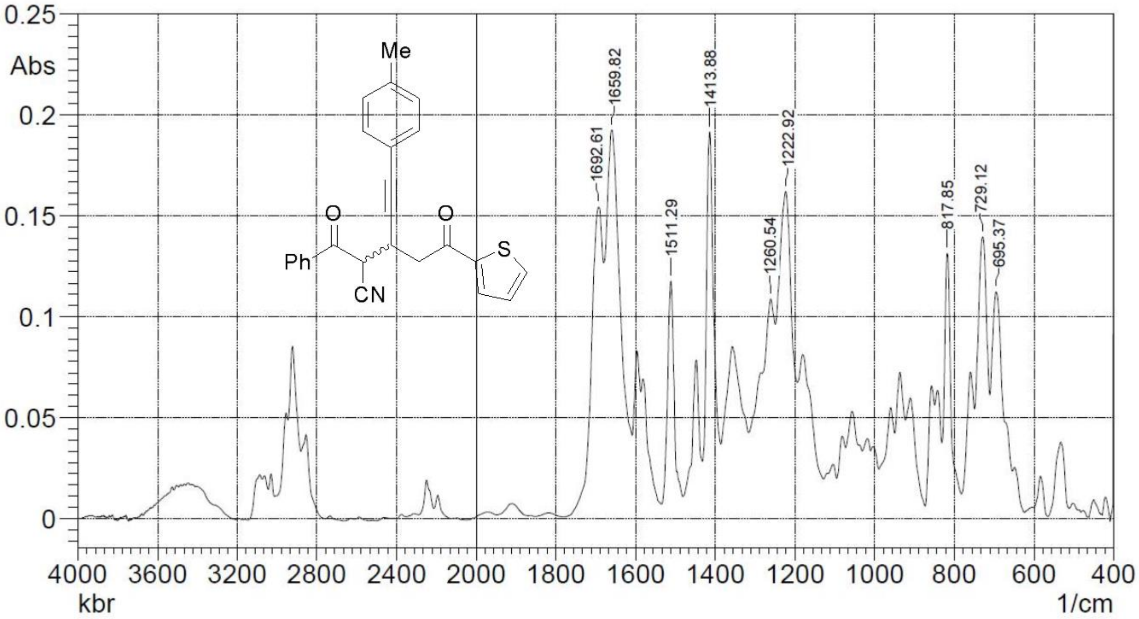

Figure S36. IR of the compound **2.1l/2.2l** (KBr).

2. ^{1}H, ^{13}C, HMQC, COSY, NOESY, HMBC, NMR spectra of compound **3.**

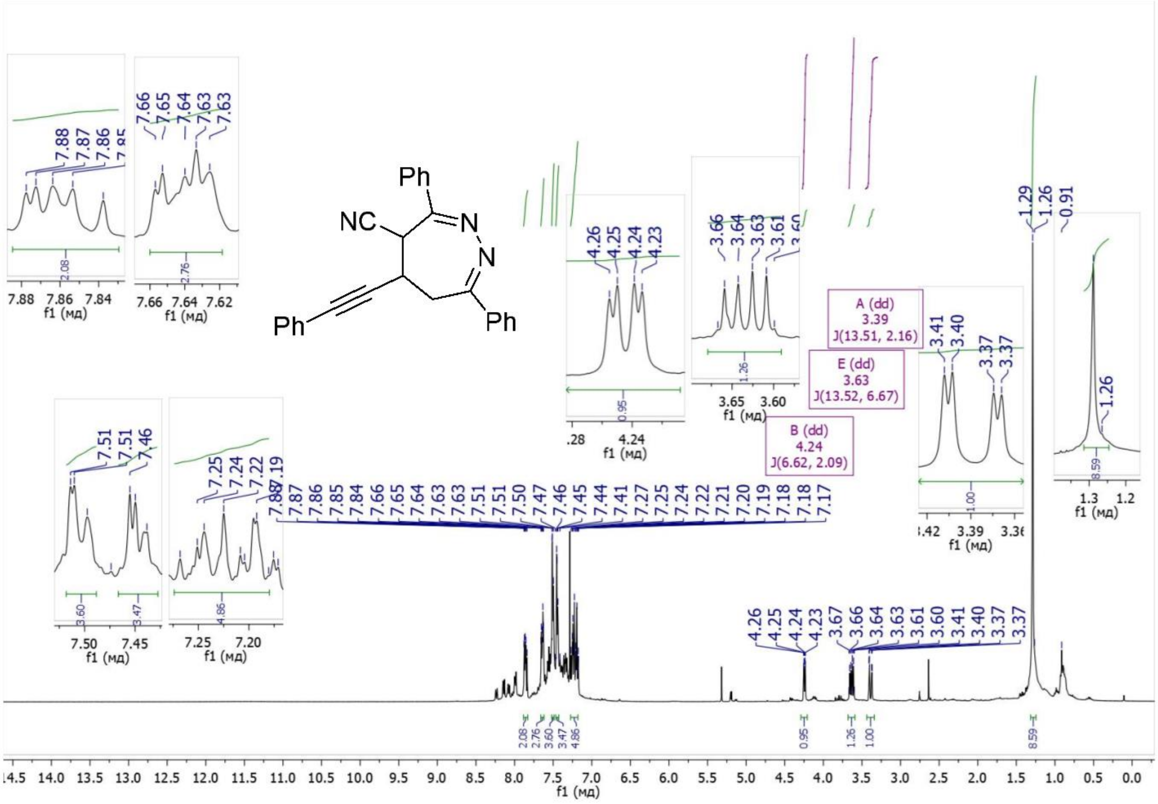

Figure S37. ^{1}H NMR spectrum of the compound **3** ($CDCl_3$, 400 MHz).

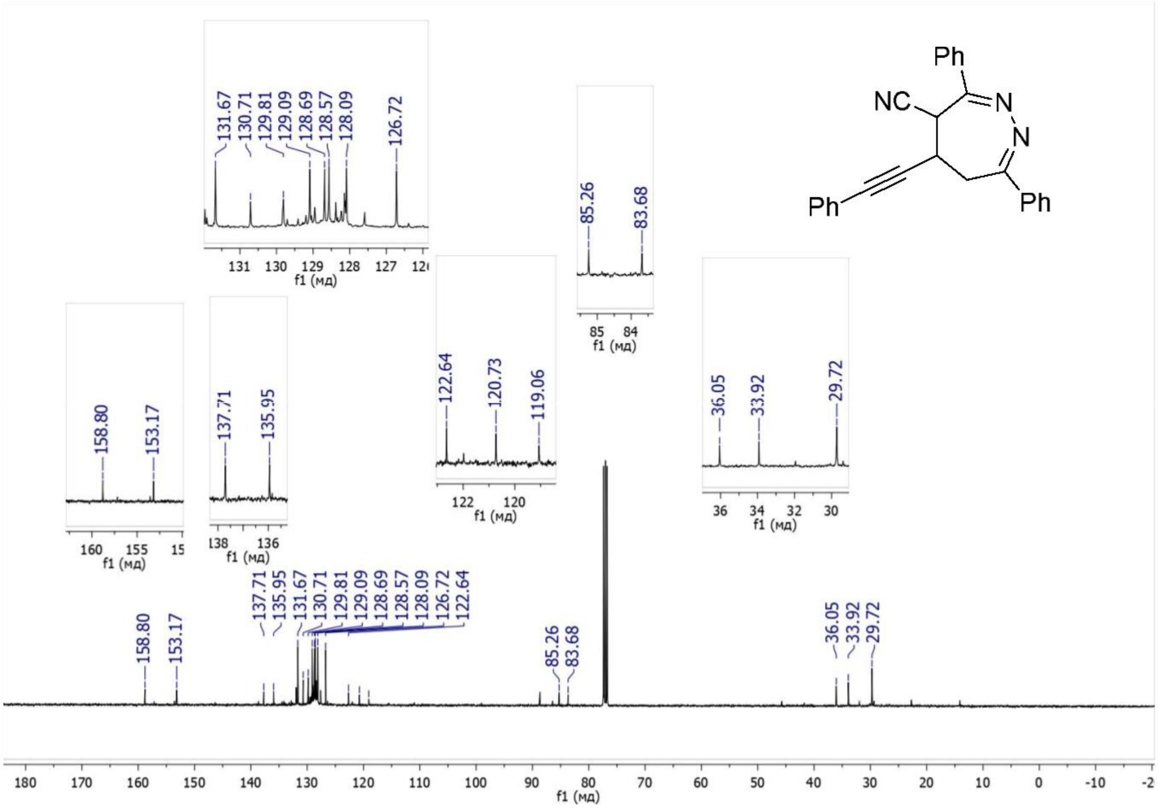

Figure S38. ^{13}C NMR spectrum of the compound **3** ($CDCl_3$, 100 MHz).

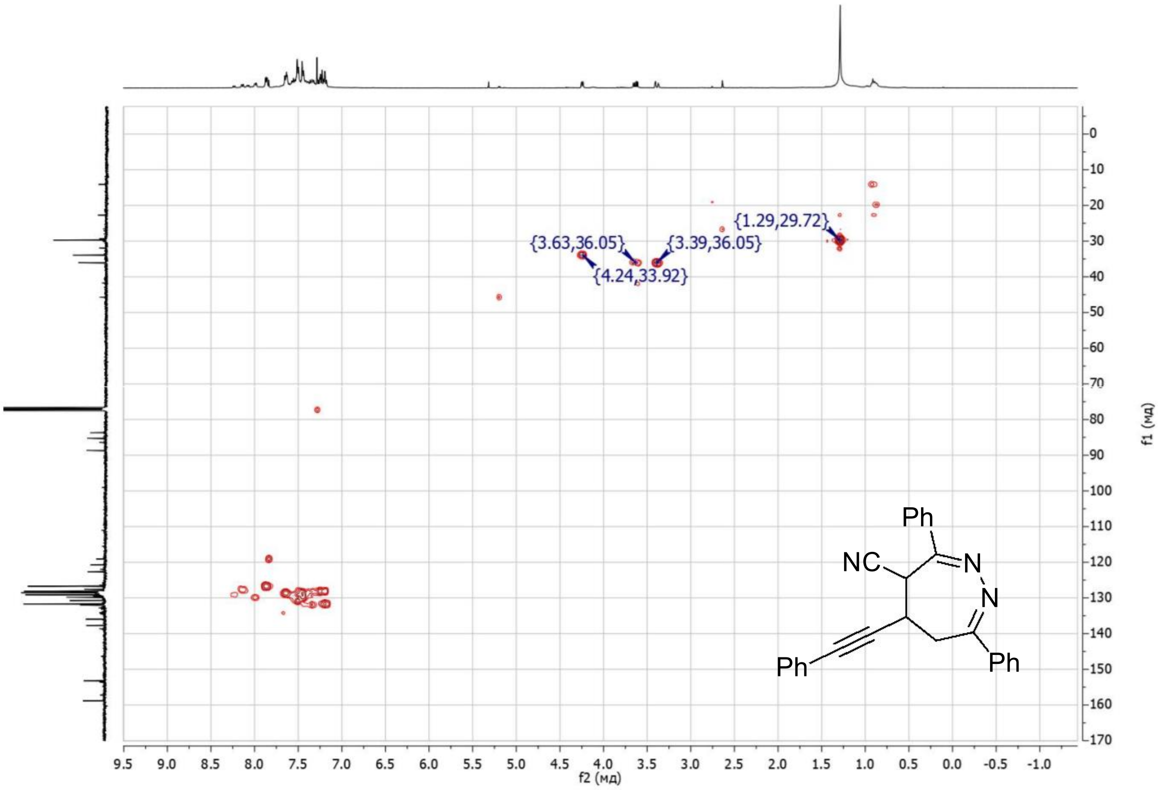

Figure S39. HMQC C-H NMR spectrum of the compound **3** ($CDCl_3$).

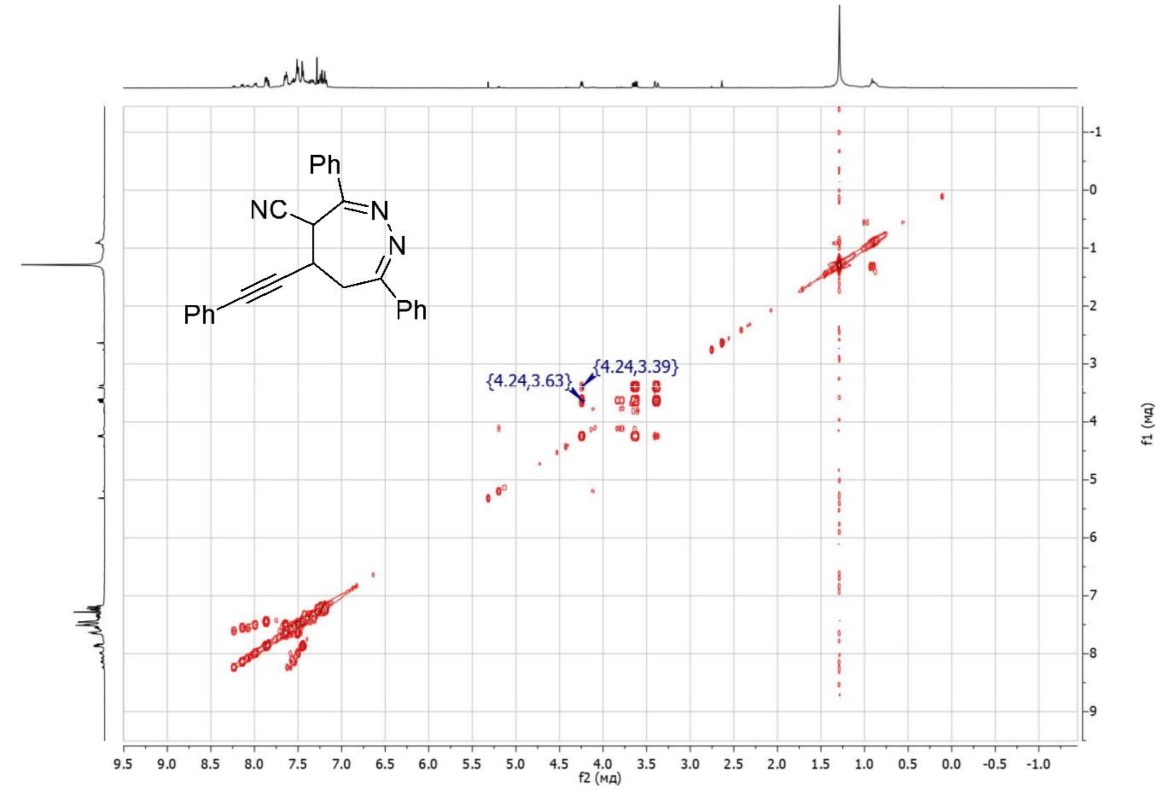

Figure S40. COSY H-H NMR spectrum of the compound **3** ($CDCl_3$).

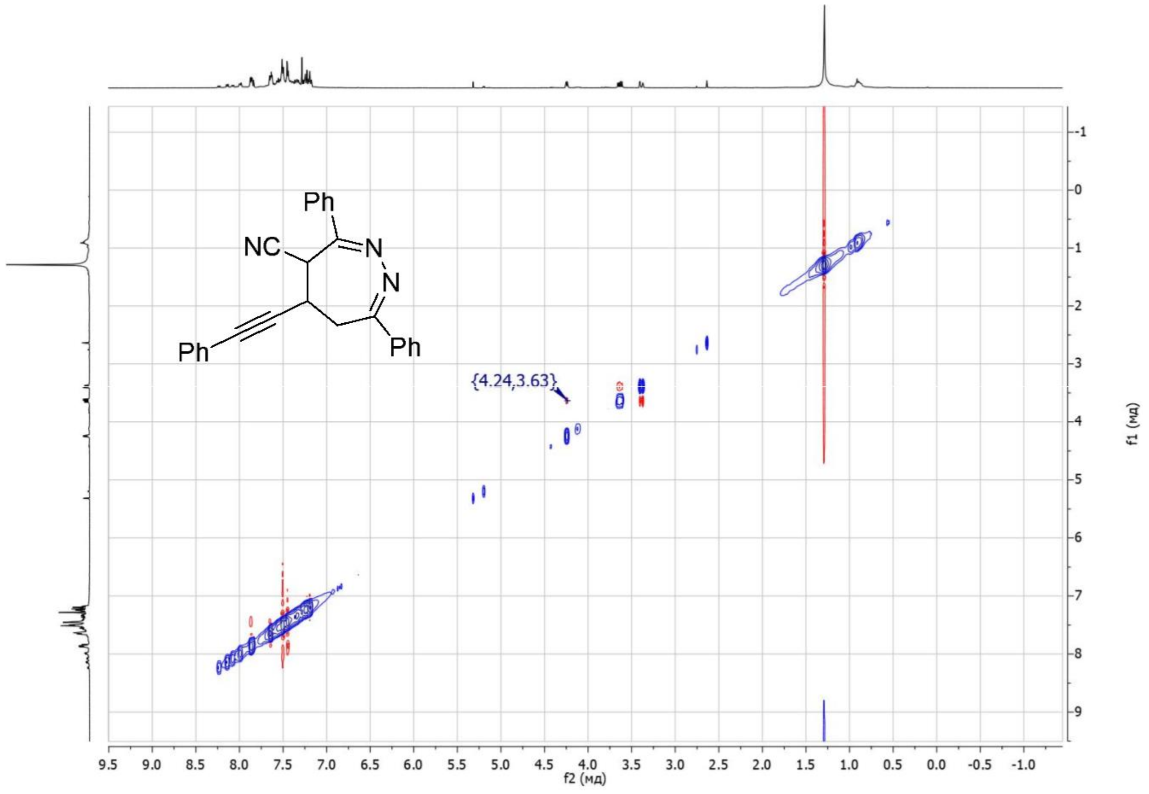

Figure S41. NOESY H-H NMR spectrum of the compound **3** ($CDCl_3$).

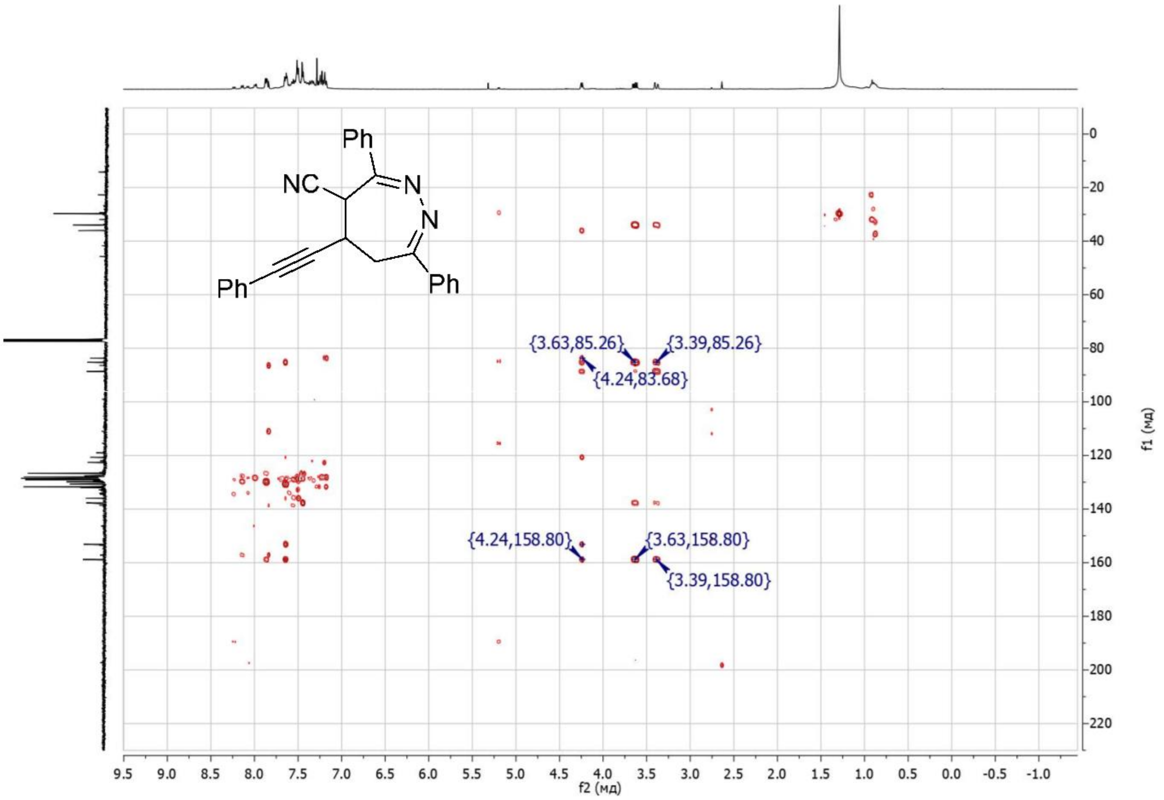

Figure S42. HMBC C-H NMR spectrum of the compound **3** ($CDCl_3$).
